# Research on optimal scheduling of integrated energy system based on improved multi-objective artificial hummingbird algorithm

**DOI:** 10.1371/journal.pone.0325310

**Published:** 2025-06-04

**Authors:** Liming Wei, Fengyang Zhang

**Affiliations:** College of Electrical and Computer Technology, Jilin Jianzhu University, Changchun, Jilin, China; SR University, INDIA

## Abstract

To accelerate energy efficiency improvement and green transition in industrial parks while addressing energy utilization and carbon reduction requirements, this study proposes a low-carbon economic dispatch model for integrated energy systems (IES) based on an enhanced multi-objective artificial hummingbird algorithm (MOAHA). The main contributions are threefold: First, we establish an optimized dispatch model incorporating combined cooling, heating and power (CCHP) systems, a refined two-stage power-to-gas (P2G) conversion process, and carbon capture technologies. Second, a stepwise carbon trading mechanism is introduced to further reduce carbon emissions from the IES. Third, a multi-strategy enhanced MOAHA is developed through three key improvements: 1) Logistic-sine fused chaotic mapping for population initialization to enhance distribution uniformity and solution quality; 2) Elite opposition-based learning and adaptive spiral migration foraging mechanisms to optimize individual positions and population diversity; 3) Simplex method integration to strengthen local search capabilities and optimization precision. Comprehensive case studies demonstrate the model’s effectiveness, achieving an 82.9% reduction in carbon emissions and 17.3% decrease in operational costs compared to conventional approaches. The proposed framework provides a technically viable solution for sustainable energy management in industrial parks, effectively balancing economic and environmental objectives.

## 1. Introduction

In response to escalating global environmental pollution and fossil energy crises, nations worldwide are increasingly prioritizing low-carbon development and establishing emission reduction targets [1]. Achieving the dual objectives of carbon peaking and carbon neutrality necessitates structural transformation and upgrading of energy systems, where decarbonizing the power sector plays a pivotal role in accelerating national carbon reduction goals [2,3]. While this transition has imposed unprecedented pressures on China’s energy structure and economic development, the synergistic evolution of integrated energy systems (IES) and renewable energy sources not only addresses these challenges but also provides innovative pathways to enhance renewable energy penetration within a low-carbon framework [4].

Integrated Energy Systems transcend the operational silos of conventional energy systems, enabling economically sustainable development while ensuring efficient and eco-friendly energy utilization [5].The multi-energy synergy inherent in IES significantly enhances energy efficiency, reduces operational costs, and mitigates carbon emissions [6].Recent advancements demonstrate diverse methodological innovations:Tan et al. developed a dispatch model for park-level IES (PIES) integrating photovoltaic/thermal (PV/T) hydrogen production with an enhanced stepped carbon trading mechanism. Their refined carbon pricing scheme effectively promoted system-wide energy conservation and emission reduction [7]. Cheng et al. emphasized that leveraging complementary interactions among heterogeneous energy carriers in multi-energy systems (MES) enables substantial carbon emission abatement [8].Luo et al. established a carbon-green certificate co-trading market framework under combined carbon emission trading (CET) and green certificate trading (GCT) mechanisms. This approach eliminates traditional market barriers while improving renewable energy accommodation rates and reducing system emissions [9].Liu et al. proposed a day-ahead dispatch model for regional IES (RIES) incorporating information gap decision theory (IGDT).By analyzing renewable output uncertainties through heat demand response (HDR)-integrated geothermal system modeling, their framework demonstrated superior cost-effectiveness and emission reduction capabilities [10].Wang et al. introduced a stepped carbon trading mechanism with incentive-penalty structures and price-signal-driven demand response strategies, effectively balancing stakeholder interests while minimizing emissions in carbon-constrained environments [11].Zhao et al. devised a two-stage distributionally robust optimization model for wind-PV-storage hybrid systems. Incorporating stepped carbon trading, their methodology reduced reliance on conventional generation units, achieving significant reductions in both emissions and economic costs while validating the model’s adaptability and superiority [12].

In recent years, heuristic algorithms have gained widespread attention and application in the optimal dispatch of IES due to their outstanding optimization performance. With their efficient solving capabilities and flexible adaptability, these algorithms have become essential tools for addressing complex optimization problems. Wang et al. developed an economic dispatch model for an electricity-heat-gas coupled regional IES incorporating a tiered carbon trading mechanism. By integrating carbon emission calculations with the carbon trading mechanism, the model incorporates carbon trading costs into the total system cost. The implementation of the fruit fly optimization algorithm demonstrates effective reduction in system operational costs [13].Considering the uncertainties of renewable energy resources, Jamal Raheela et al. proposed a scenario reduction technique based on an enhanced artificial hummingbird algorithm integrated with Monte Carlo simulation. This method generates an appropriate number of scenarios through Monte Carlo simulation reduction and employs the enhanced artificial hummingbird algorithm to solve both stochastic and deterministic optimal reactive power dispatch (ORPD) problems. Simulation results demonstrate that the enhanced artificial hummingbird algorithm effectively reduces active power losses, improves voltage profiles, and enhances voltage stability when addressing ORPD challenges [14]. Kou et al. addressed the susceptibility of wind turbines to failure in harsh offshore wind farm environments by proposing a chaotic simulated annealing genetic algorithm (CSAGA) incorporating asymmetric time considerations. The improved algorithm integrates logistic-tent chaotic mapping with genetic algorithms and simulated annealing techniques. Comparative simulation experiments demonstrated the superior performance of CSAGA over alternative algorithms [15]. Guo et al. developed a novel multi-strategy adaptive artificial bee colony algorithm (MSABC) for solving complex optimization problems. The MSABC incorporates an evolutionary rate index and an innovative elite-guided search strategy. Experimental results comparing MSABC with existing algorithms confirmed its enhanced convergence performance [16]. Ramadan Ashraf et al. accounted for uncertainties in load demand and renewable distributed generator (RDG) output power by proposing an efficient state-of-the-art technique for optimal RDG sizing and placement in radial distribution systems. The implementation of the artificial hummingbird algorithm (AHA) effectively mitigated power losses, reduced voltage deviation, and minimized expected costs [17].

However, single-objective optimization algorithms, constrained by their singular optimal solution output, inherently obscure the intrinsic conflicts and synergies among multidimensional objectives (e.g., economic efficiency and environmental sustainability) while oversimplifying the complexity of multi-objective synergistic optimization. This simplification risks convergence to local optima, hindering globally efficient resource allocation across multidimensional systems. In contrast, multi-objective optimization algorithms overcome these limitations via parallel optimization mechanisms, simultaneously addressing competing objectives without predefined weightings. By generating a Pareto-optimal solution set, they explicitly quantify trade-off relationships between goals such as economic viability and low-carbon performance, thereby eliminating biases from artificial intervention. The diverse non-dominated solutions within the set empower decision-makers with adaptable strategies spanning cost-prioritized to emission-reduction-focused approaches, accommodating dynamic scenario-specific demands. Furthermore, their global search mechanisms circumvent local optima traps, enabling synergistic resource utilization within multidimensional trade-off spaces. Owing to their superior alignment with complex and evolving optimization paradigms, multi-objective optimization algorithms have garnered substantial research attention in recent years [18].Shan et al. developed an energy optimization model that successfully achieves a globally optimal configuration using an enhanced multi-objective gray wolf algorithm [19]. Wu et al. proposed a grid-connected IES that considers the complementarity between geothermal energy, solar energy, and heat storage. A multi-objective non-dominated sorting genetic algorithm was introduced to optimize the system, improving operational efficiency [20]. Wang et al. constructed a dual-level optimization model for regional IES, considering both the quantity and quality of energy. By embedding a tabu search algorithm into a multi-objective genetic algorithm, they were able to effectively solve the model and significantly reduce the system’s economic cost [21]. Li et al. addressed complex models with multi-objective, non-convex, and strongly constrained decision variables by proposing a multi-objective whale optimization algorithm to solve a two-stage robust game model [22].

In summary, multi-objective optimization algorithms offer a critical pathway for addressing the synergistic optimization of multidimensional objectives in integrated energy systems. By generating a Pareto-optimal solution set, these algorithms explicitly quantify trade-off relationships among competing objectives, providing decision-makers with diverse strategy options and significantly enhancing system flexibility in dynamic scenarios. However, existing algorithms still face challenges such as local optima entrapment and slow convergence rates, particularly in complex systems with high-dimensional, nonlinear constraints, where traditional multi-objective methods often exhibit insufficient global search capabilities, leading to degraded solution quality, prolonged optimization time, and compromised real-time scheduling efficacy. To address these limitations, this study proposes a multi-strategy enhanced multi-objective artificial hummingbird algorithm for low-carbon economic dispatch. The model integrates adaptive spiral migration foraging and simplex method strategies to simultaneously avoid local optima and accelerate convergence. The research contributions are threefold:

(1) A low-carbon economic dispatch optimization model is developed, minimizing total system costs while balancing economic and carbon emission objectives through the coordinated operation of carbon capture, refined power-to-gas processes, and a stepwise carbon trading mechanism.
(2) A multi-strategy enhanced multi-objective artificial hummingbird algorithm is proposed, integrating logistic-sine fused chaotic mapping, elite opposition-based learning, adaptive spiral migration foraging, and simplex method strategies. The logistic-sine fused chaotic mapping and elite opposition-based learning enhance population initialization by improving the uniformity of initial populations and the quality of initial solutions, while adaptive spiral migration foraging and the simplex method refine overall population quality by updating positions of individuals with the lowest fitness, thereby strengthening the algorithm’s local search capability and optimization efficiency.
(3) Simulation experiments validate the effectiveness of the proposed method. The improved multi-objective artificial hummingbird algorithm effectively addresses the challenges of local optima traps and slow convergence in high-dimensional objective spaces when applied to complex multi-dimensional problems in integrated energy systems, providing a reliable technical solution for achieving the “dual-carbon” targets in such systems.

## 2. Optimal scheduling model of integrated energy system in industrial park

### 2.1 Integrated energy system model

**Fig 1:**
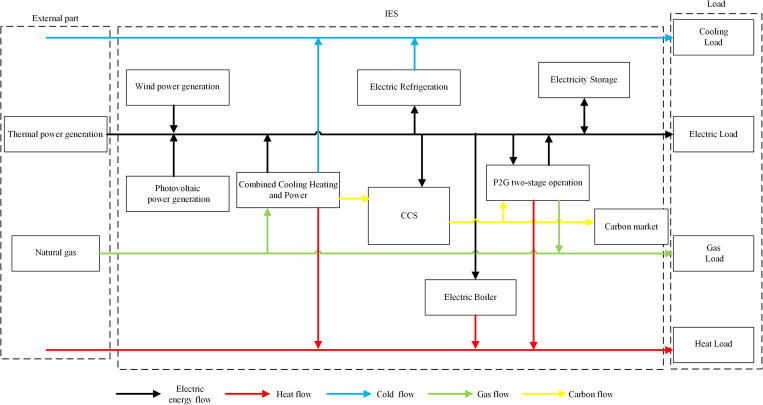
Industrial park integrated energy system model.

The comprehensive energy system can effectively integrate and coordinate the operation of a variety of energy facilities to achieve synergistic and complementary power supply, so as to enhance the economic benefits of the entire energy system, but also better adapt to the diversified and multi-form energy consumption needs of modern society. In an Integrated Energy System, when a Combined Cooling, Heating, and Power unit equipped with carbon capture technology operates, the byproduct CO₂ is supplied as feedstock to Power-to-Gas equipment. The hydrogen produced by electrolyzers reacts with CO₂ through methanation to generate CH₄, which is then delivered to gas loads via the natural gas pipeline. The CCHP unit, serving as a coupling device for electrical, thermal, and cooling subsystems, provides electrical, heating, and cooling loads to users within the system. The thermal energy generated by electric boilers is utilized to meet the thermal load demand of users.The cold energy generated by electric refrigeration is used to meet the cooling load requirements of users. The integrated energy system model of industrial park is shown in Fig 1.

### 2.2 Model of combined cooling, heating and electricity supply unit

**Fig 2:**
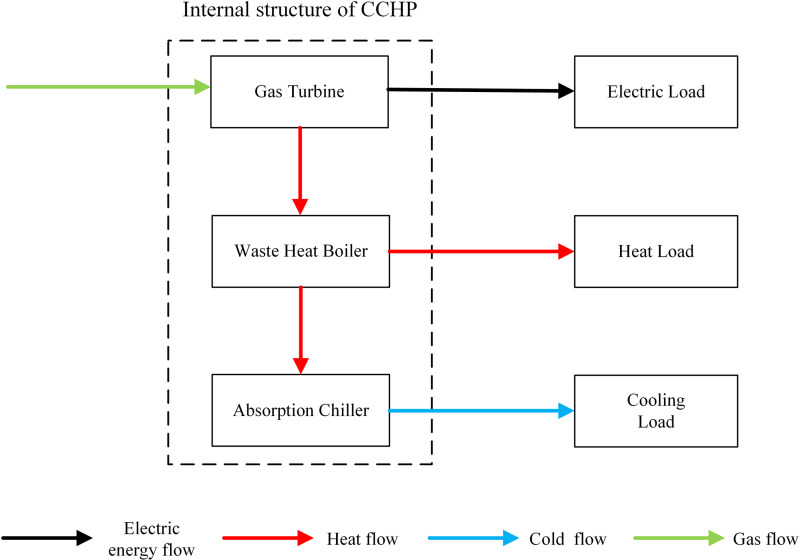
Internal structure of combined cooling, heat and electricity supply.

Combined cooling, heating, and power (CCHP) is a high-efficiency energy system that generates electricity via gas turbines while recovering waste heat from the power generation process for space heating or to drive absorption chillers for cooling, thereby achieving tri-generation of electricity, heat, and cold, with its internal configuration illustrated in Fig 2.

The model of the combined cooling, heating, and power supply unit is presented in Equation (1).


$$\begin{array}{c} \left\{ \begin{array}{c} R_{WHB}(t)=\eta_{H}\frac{P_{CCHP}(t)}{\eta_{e}}\left(1-\eta_{e}-\eta_{loss}\right)\\ C_{AC}(t)=R_{WHB}(t)\frac{\eta_{c}}{\eta_{c}+\eta_{h}}\\ P_{CCHP}(t)={\eta_{e}Q}_{CCHP}(t) \end{array}\right.\; \end{array}$$ (1)

Where $R_{WHB}(t)$ is the heat power output of the waste-heat boiler at time *t*; $\eta_{H}$ is the heat loss coefficient; $P_{CCHP}(t)$ is *t*he electrical power output of the CCHP unit; $\eta_{e}$ is the power generation efficiency of the CCHP unit; $\eta_{loss}$ is the waste-heat loss efficiency; $C_{AC}(t)$ is the cold power output of the absorption refrigerator${;\;\eta }_{c}$ is the CCHP refrigeration efficiency; $\eta_{h}$ is the heat production efficiency of the CCHP; $Q_{CCHP}(t)$ is the gas power consumed by the CCHP unit.

### 2.3 Carbon capture model

Carbon capture systems are engineered installations that extract CO₂emissions from industrial exhaust streams or energy production processes, preventing direct atmospheric release through integrated compression followed by either geological sequestration or conversion into industrial feedstocks, thereby achieving substantial greenhouse gas emission reductions; in the power generation sector, the integrated application of carbon capture and storage (CCS) technology plays a pivotal role in enhancing decarbonization effectiveness, making the retrofitting of conventional power plants with carbon capture systems a critical development trend in coming years [23], while the captured CO₂can additionally serve as a carbon source for power-to-gas (P2G) conversion processes [24].

A carbon capture device is installed in the CCHP unit. The electrical power output of the CCHP unit is composed of two parts: (1) the power consumption of the carbon capture device, $P_{CCS,e}(t)$, and (2) the remaining power consumption, referred to as the net output power of the CCHP unit, $P_{CCHP,e}(t)$. The total electrical power output $P_{CCHP}(t)$ of the CCHP unit at time *t* is given by Equation (2).


$$P_{CCHP}(t)=P_{CCS,e}(t)+P_{CCHP,e}(t)$$ (2)

The total energy consumption power of the carbon capture device consists of two parts: (1) the basic energy consumption power $P_{CCS,b}(t)$, which can be considered a constant, and (2) the operational energy consumption power $P_{CCS,r}(t)$, which is related to the amount of CO_2_ captured during operation. This relationship is represented by Equation (3).


$$\begin{array}{c} \left\{ \begin{array}{c} \begin{array}{c} P_{CCS,e}(t)=P_{CCS,b}(t)+P_{CCS,r}(t)\\ P_{CCS,r}(t)=\lambda_{CCS}\eta_{CCS}P_{CCHP,ca}(t)\\ P_{CCHP,ca}(t)=e_{c}P_{CCHP}(t) \end{array}\\ C_{ca}(t)=\eta_{CCS}P_{CCHP,ca}(t) \end{array}\right. \end{array}$$ (3)

Where $P_{CCHP,ca}(t)$ is the carbon emission of the CCHP unit; $\lambda_{CCS}$ is the energy consumption for CO₂ capture; $\eta_{CCS}$ is the carbon capture efficiency; $e_{c}$ is the unit carbon emission intensity; and $C_{ca}(t)$ is the amount of CO_2_ captured by the CCHP unit.

### 2.4 Power to gas two-stage operation model

**Fig 3:**
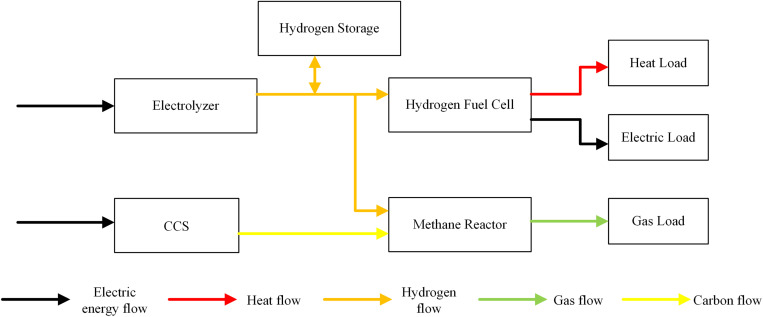
P2G two-stage operation process.

Power-to-gas (P2G) systems, serving as critical bidirectional coupling interfaces between electrical grids and natural gas networks, have garnered increasing research attention in IES due to their system flexibility enhancement potential [25]. The two-stage P2G conversion process enables efficient transformation of surplus renewable electricity into chemically storable and pipeline-transportable synthetic natural gas through sequential technological pathways: (1) hydrogen production via proton exchange membrane electrolysis and (2) subsequent catalytic methanation by reacting the produced hydrogen with captured carbon dioxide [26], with the complete technical schematic of this process illustrated in Fig 3.

The electrolyzer converts surplus electrical energy into hydrogen through water electrolysis, with its mathematical model represented by Equation (4).


$$P_{EL,H}(t)=\eta_{EL}P_{EL,e}(t)$$ (4)

Where $P_{EL,H}(t)$ is the hydrogen generation power of the EL device; $\eta_{EL}$ is the EL conversion efficiency; $P_{EL,e}(t)$ is the electrical power consumption of the EL device.

The hydrogen fuel cell (HFC) is an electrochemical device that directly converts the chemical energy of hydrogen and oxygen into both electrical energy and thermal energy, with its mathematical formulation presented in Equation (5).


$$\begin{array}{c} \left\{ \begin{array}{c} R_{HFC}(t)=\eta_{HFC,h}P_{HFC}(t)\\ P_{HFC,e}(t)=\eta_{HFC,e}P_{HFC}(t) \end{array}\right.\; \end{array}$$ (5)

Where $R_{HFC}(t),\;P_{HFC,e}(t)$ are the output thermal power of the HFC and the electrical power output of the HFC, respectively; $P_{HFC}(t)$ is the input hydrogen power for the HFC; $\eta_{HFC,h}$, $\eta_{HFC,e}$ are the efficiencies of heat and electricity conversion, respectively.

The methanation reactor (MR) catalytically synthesizes methane through the Sabatier reaction between hydrogen produced from water electrolysis and captured carbon dioxide, with its governing equations mathematically formulated in Equation (6).


$$\begin{array}{c} \left\{ \begin{array}{c} Q_{MR}(t)=\eta_{MR}P_{MR}(t)\\ M_{{CO}_{2}}(t)=\rho_{{\text{CO}}_{2}}V_{{CO}_{2}}(t)\\ V_{{CH}_{4}}(t)=V_{{CO}_{2}}(t)\\ V_{{CH}_{4}}(t)=\frac{R_{EH}\eta_{MR}P_{MR}(t)}{R_{{CH}_{4}}} \end{array}\right.\; \end{array}$$ (6)

Where $Q_{MR}(t)$ is the gas power output of MR; $\eta_{MR}$ is MR conversion efficiency; $P_{MR}(t)$ is MR input hydrogen power; $M_{{CO}_{2}}(t)$ is the amount of CO_2_ consumed; $\rho_{{\text{CO}}_{2}}$ is the density of CO_2_; $V_{{CO}_{2}}(t)$ and $V_{{CH}_{4}}(t)$ are the volumes of CO_2_ consumed and methane produced, respectively; $R_{EH}$ is the conversion coefficient of electricity generation into heat supply; and $R_{{CH}_{4}}$ is the calorific value of natural gas combustion.

### 2.5 Electric refrigerator model

The model for the electric refrigerator (ER) is given in Equation (7).


$$C_{ER}(t)=\eta_{ER}P_{ER}(t)$$ (7)

Where $C_{ER}(t)$ is the output cold power of the ER; $\eta_{ER}$ is the refrigeration coefficient of the ER; $P_{ER}(t)$ is the electrical power consumed by the ER.

### 2.6 Electric boiler model

The electric boiler model is given by Equation (8).


$$R_{EB}(t)=\eta_{EB}P_{EB}(t)$$ (8)

Where $R_{EB}(t)$ is the output thermal power of the electric boiler; $\eta_{EB}$ is the conversion efficiency of the electric boiler; and $P_{EB}(t)$ is the electric power consumed by the electric boiler.

### 2.7 Energy storage model

The models of different types of energy storage devices in an integrated energy system are similar. In this paper, the two types of energy storage devices are unified, as shown in Equation (9).


$$E_{ES,i}(t)={(1-\Delta t)E}_{ES,i}(t-1)+\eta_{i}^{\text{cha}}P_{i}^{cha}(t)-\;P_{i}^{dis}(t)/\eta_{i}^{dis}$$ (9)

Where $E_{ES,i}(tand\;E_{ES,i}(t-1)$ represent the energy storage capacity of the *i* type energy storage device at times t and t-1; Δt is the energy loss rate of *t*he *i t*ype energy storage device; $\eta_{i}^{\text{cha}}\;and\;\eta_{i}^{dis}$ are the charging and discharging efficiencies of the *i* type energy storage device; $P_{i}^{cha}(tand\;P_{i}^{dis}(t)$ are the charging and discharging power of the *i* type energy storage device at time t.

## 3. Modeling of cascade carbon trading mechanism

To achieve the dual objectives of carbon neutrality and peak carbon emissions, carbon trading markets are undergoing rapid development, with two predominant trading mechanisms emerging: conventional carbon trading and stepped carbon trading. Comparative analyses demonstrate that stepped carbon trading mechanisms exhibit superior efficacy in both emissions control and mitigation promotion relative to traditional approaches [27]. The stepped mechanism establishes a nonlinear relationship between carbon emission costs and emission volumes through phased pricing tiers and dynamic quota adjustments, operating via a structured framework: (1) initial quota allocation based on sector-specific emission caps, followed by (2) mandatory purchase of additional allowances at progressively increasing price tiers when emissions exceed allocated quotas, thereby creating market-driven resource reallocation that reduces aggregate societal abatement costs [28].

The actual carbon emission model is given by Equation (10).


$$\begin{array}{c} \left\{ \begin{array}{c} E_{IES}=E_{CCHP}+E_{e,buy}-E_{CCS}\\ E_{CCHP}=r^{c}\sum_{t=1}^{T}P_{CCHP}(t)\\ E_{e,buy}=r^{e}\sum_{t=1}^{T}P_{e,buy}(t)\\ E_{CCS}=\sum_{t=1}^{T}C_{ca}(t) \end{array}\right.\; \end{array}$$ (10)

Where $E_{IES}$ is the actual carbon emission of IES; $E_{CCHP}$ is the actual carbon emission from the CCHP units; $E_{e,buy}$ is the actual carbon emissions associated with electricity purchased from the grid; $E_{CCS}$ is the total amount of CO_2_ captured by the carbon capture devices; $r^{c}$ is the carbon emissions generated by the CCHP units; $r^{e}$ is the carbon emission per unit of electricity generation; $P_{e,buy}(t)$ is the electric power purchased from the grid; *T* represents a time period, typically 24 h.

The carbon emission allowance quota model is shown in Equation (11).


$$\begin{array}{c} \left\{ \begin{array}{c} D_{IES}=D_{CCHP}+D_{e,buy}\\ D_{CCHP}=\lambda^{c}\sum_{t=1}^{T}P_{CCHP}(t)\\ D_{e,buy}=\lambda^{e}\sum_{t=1}^{T}P_{e,buy}(t) \end{array}\right.\; \end{array}$$ (11)

Where $D_{IES}$ is the carbon emission quota for the IES; $D_{CCHP}$ is the initial free carbon emission quota for the CCHP units; $D_{e,buy}$ is the initial free carbon emission quota for electricity purchased from the grid; $\lambda^{c}$ is the carbon emission quota per unit of electricity generated by the CCHP unit; and $\lambda^{e}$ is the carbon emission quota per unit of electricity purchased from the grid.

Step carbon trading mechanism model

The stepped carbon trading mechanism divides the carbon emission trading volume into several intervals. The carbon trading price increases progressively with higher carbon emissions. Additionally, a compensation coefficient is introduced so that when the carbon emission allowance exceeds actual emissions, the excess carbon emission allowance can be sold. This is represented by Equations (12) and (13).


$$F_{IES}=E_{IES}-D_{IES}$$ (12)

$$\begin{array}{c} f_{price}^{{CO}_{2}}=\left\{ \begin{array}{c} \alpha (1+3g)\left(F_{IES}+2l\right)-\alpha (2+3g)l,\\ F_{IES}\leq -2l\\ \alpha (1+2g)\left(F_{IES}+l\right)-\alpha (1+g)l,\\ -2l<F_{IES}\leq -l\\ \alpha (1+g)F_{IES},\\ -l\leq F_{IES}\leq 0\\ \alpha F_{IES},\\ F_{IES}\leq l\\ \alpha (1+\tau )\left(F_{IES}-l\right)+\alpha l,\\ l<F_{IES}\leq 2l\\ \alpha (1+2\tau )\left(F_{IES}-2l\right)+\alpha (2+\tau )l,\\ F_{IES}\geq 2l \end{array}\right.\; \end{array}$$ (13)

Where $F_{IES}$ is the total carbon emissions in the stepped carbon trading mechanism; $\;f_{price}^{{CO}_{2}}$ is the cost of step-type carbon trading; α is the base price of the cascade carbon trading; g is the compensation coefficient; l is the length of each carbon emission interval in the cascade carbon trading; τ is the growth rate of the step carbon trading price.

## 4. Objective functions and constraints

### 4.1 Objective function

In this paper, we define the comprehensive cost of the system, denoted as $F_{1}$, and carbon emissions, represented by $F_{2}$, as our objective functions. The comprehensive cost includes various components such as electricity purchase costs ($f_{e,buy}$), gas purchase costs ($f_{g,buy}$), and operational expenses ($f_{p}$), as illustrated in Equation (14).


$$\begin{array}{c} \left\{ \begin{array}{c} F_{1}=min{(f}_{e,buy}+f_{g,buy}+f_{p})\\ F_{2}=F_{IES} \end{array}\right.\; \end{array}$$ (14)

The cost of purchasing electricity is given by Equation (15).


$$f_{e,buy}=\sum_{t=1}^{T}\sigma^{e}P_{e,buy}(t)$$ (15)

Where $\sigma^{e}$ is the electricity purchase price; $P_{e,buy}(t)$ is the amount of electricity purchased from the grid.

The gas purchase cost is expressed in Equation (16).


$$f_{g,buy}=\sum_{t=1}^{T}\sigma^{g}P_{g,buy}(t)$$ (16)

Where $\sigma^{g}$ is the gas purchase price;$P_{g,buy}(t)$ is the amount of gas purchased.

The operating costs are shown in Equation (17).


$$\begin{array}{c} f_{p}=\sum_{t=1}^{T}\left(\begin{array}{c} C_{PV}P_{PV}(t)+C_{WT}P_{WT}(t)+C_{CCHP}P_{CCHP}(t)+C_{WHB}R_{WHB}(t)+C_{ac}C_{AC}(t)+C_{EB}P_{EB}(t)\\ {+C}_{CCS}P_{CCS,e}(t)+C_{EL}P_{EL,e}(t)\;+C_{MR}P_{MR}(t)+{C_{HFC}P}_{HFC}(t)+C_{ER}P_{ER}(t) \end{array}\right) \end{array}$$ (17)

Where $C_{PV}$*,* $C_{WT},\;C_{CCHP},\;C_{WHB},\;C_{ac},\;C_{CCS},\;C_{EL},\;C_{MR},\;C_{HFC},\;C_{EB},\;and\;C_{ER}$ are the operation and maintenance cost coefficients for the respective equipment. The detailed equipment parameters are provided in Table 1.

**Table 1:** Equipment parameters.

Argument	Operation and maintenance cost (Yuan/KW•h)	Argument	Operation and maintenance cost (Yuan/KW•h)
$C_{ER}$	0.02	$C_{CCS}$	0.055
$C_{EB}$	0.016	$C_{CCHP}$	0.09
$C_{WHB}$	0.01	$C_{PV}$	0.024
$C_{WT}$	0.0196	$C_{ac}$	0.06

### 4.2 Constraint condition

The electrical power balance constraint is given by Equation (18).


$$P_{WT}(t)+P_{PV}(t)+P_{CCHP}(t)+P_{e,buy}(t)+P_{e}^{dis}(t)+P_{HFC,e}(t)=P_{EL,e}(t)+P_{EB}(t)+P_{ER}(t)+P_{e}^{cha}(t)+P_{e,load}(t)$$ (18)

Where $P_{WT}(tand\;P_{PV}(t)$ are the electrical power output from wind power and the electrical power output from photovoltaic systems, respectively; $P_{e,load}(t)$ is the electrical power demand; $P_{e}^{cha}(tand\;P_{e}^{dis}(t)$ are the energy storage charging power and the energy storage discharging power, respectively.

The thermal power balance constraint is given by Equation (19).


$$R_{WHB}(t)+R_{EB}(t)+R_{HFC}(t)=R_{h,load}(t)$$ (19)

Where $R_{h,load}(t)$ is the thermal power demand.

The cold power balance constraint is given by Equation (20).


$$C_{AC}(t)+C_{ER}(t)=C_{c,load}(t)$$ (20)

Where $C_{c,load}(t)$ is the cold power demand.

The gas power balance constraint is given by Equation (21).


$$P_{g,buy}(t)+Q_{MR}(t)=Q_{CCHP}(t)+Q_{g,load}(t)$$ (21)

Where $Q_{g,load}(t)$ is the gas power demand.

The hydrogen power balance constraints are given by Equation (22).


$$P_{EL,H}(t)+P_{H}^{dis}(t)=P_{MR}(t)+P_{HFC}(t)+P_{H}^{cha}(t)$$ (22)

Where $P_{H}^{cha}(t)$ and $P_{H}^{dis}(t)$ are the energy storage charging power of the hydrogen storage device and the energy storage discharging power of the hydrogen storage device, respectively.

The constraints for the CCHP units are shown in Equation (23).


$$\left\{ \begin{array}{c} 0\leq P_{CCHP}(t)\leq P_{CCHP}^{\max}(t)\\ 0\leq R_{WHB}(t)\leq R_{WHB}^{\max}(t)\\ 0\leq C_{AC}(t)\leq C_{AC}^{\max}(t) \end{array}\right.\;$$ (23)

Where $P_{CCHP}^{\max}(t)$*,* $R_{WHB}^{\max}(t)$*,* and $C_{AC}^{\max}(t)$ are the upper limit of the electrical, thermal, and cold power output of the CCHP unit, respectively.

The constraints for the ER are given by Equation (24).


$$0\leq C_{ER}(t)\leq C_{ER}^{\max}(t)$$ (24)

Where $C_{ER}^{\max}(t)$ is the upper limit of the cold power output from the ER.

The constraints for the CCS are shown in Equation (25).


$$0\leq P_{CCS,e}(t)\leq P_{CCS,e}^{\max}(t)$$ (25)

Where $P_{CCS,e}^{\max}(t)$ is the upper limit of the electrical power consumption of the CCS.

The constraint for the EL is shown in Equation (26).


$$0\leq P_{EL,e}(t)\leq P_{EL,e}^{\max}(t)$$ (26)

Where $P_{EL,e}^{\max}(t)$ is the upper limit of the electrical power consumed by the EL.

The MR constraint is given by Equation (27).


$$0\leq P_{MR}(t)\leq P_{MR}^{\max}(t)$$ (27)

Where $P_{MR}^{\max}(t)$ is the upper limit of the hydrogen power input for the MR.

The constraints for the HFC are shown in Equation (28).


$$0\leq P_{HFC}(t)\leq P_{HFC}^{\max}(t)$$ (28)

Where $P_{HFC}^{\max}(t)$ is the upper limit of the hydrogen power input for the HFC.

The EB constraint is shown in Equation (29).


$$0\leq R_{EB}(t)\leq R_{EB}^{\max}(t)$$ (29)

Where $R_{EB}^{\max}(t)$ is the upper limit of the thermal power output of the EB.

The operation constraints for controllable units are given by Equation (30).


$$\begin{array}{c} \left\{ \begin{array}{c} P_{i}^{\min}\leq P_{i}(t)\leq P_{i}^{\max}\\ {CL}_{i}^{\min}\leq P_{i}(t)-P_{i}(t-1)\leq {CL}_{i}^{\max} \end{array}\right.\; \end{array}$$ (30)

Where $P_{i}(t)$ is the output of the *i* controlled unit at time *t*; $P_{i}^{\min}$ and $P_{i}^{\max}$ are the minimum and maximum output limi*t*s of the *i* controlled unit; ${CL}_{i}^{\min}$ and ${CL}_{i}^{\max}$ are the lower and upper limits of the climbing speed for the *i* controlled unit.

The energy storage constraints are given by Equation (31).


$$\begin{array}{c} \left\{ \begin{array}{c} \lambda_{i}^{\min}Z_{\text{ES,}i}\leq E_{\text{ES,i}}(t)\leq \lambda_{i}^{\max}Z_{\text{ES,}i}\\ {-\Delta }_{i}^{cha}P_{\text{ES,i}}^{\text{min}}\leq P_{\text{ES,i}}(t)\leq \Delta_{i}^{dis}P_{\text{ES,i}}^{\text{max}}\\ \mu_{i}^{cha}(t)\times \mu_{i}^{dis}(t)=0\\ E_{\text{ES,i}}(1)=E_{\text{ES,i}}(T)\\ E_{\text{ES,i}}^{\min}\leq E_{\text{ES,i}}\leq E_{\text{ES,i}}^{\max} \end{array}\right.\; \end{array}$$ (31)

Where $\lambda_{i}^{\min}$ and $\lambda_{i}^{\max}$ are the minimum and maximum charge states of the *i* type energy storage device; $Z_{\text{ES,i}}$ is the total capacity of the *i* type energy storage device; $\Delta_{i}^{cha}$ and $\Delta_{i}^{dis}$ are the maximum charging and discharging efficiencies of the *i* type energy storage device; $P_{\text{ES,i}}^{\text{min}}$ and $P_{\text{ES,i}}^{\text{max}}$ are the lower and upper power limits of the *i* type energy storage device; $\mu_{i}^{cha}(t)$ and $\mu_{i}^{dis}(t)$ are the state variables of energy storage and discharge for the *i* type energy storage device at time t; $E_{\text{ES,i}}^{\min}$ and $E_{\text{ES,i}}^{\max}$ are the lower and upper limits of the capacity of the *i* type energy storage device.

## 5. Improved multi-objective artificial hummingbird algorithm

### 5.1 Artificial hummingbird algorithm

The Artificial Hummingbird Algorithm (AHA), proposed by Zhao et al. (2022) as a novel metaheuristic optimization technique, mimics the unique flight capabilities and intelligent foraging strategies observed in natural hummingbirds to achieve superior search performance [29]. Distinguished from conventional metaheuristics, AHA demonstrates three salient characteristics: (1) minimal parameter requirements, (2) stable convergence rates, and (3) enhanced optimization capacity, with its bio-inspired framework – particularly the emulation of hummingbirds’ specialized search and nectar exploration mechanisms – conferring distinct advantages in solving specific optimization challenges [30]. The algorithm computationally replicates hummingbird foraging behavior through an innovative visitation table system that implements memory functions by: 1) recording food source quality grades, 2) determining visitation priority levels based on elapsed time since last visit (longer intervals yielding higher priority), and 3) selecting targets with either the highest priority level or, when levels are equal, the maximal nectar replenishment rate (quantified by current fitness values).

#### Initial population.

Place N hummingbirds on each food source, and initialize their positions as defined in Equation (32):


$$x_{i}=LB+r\times (UB-LB),i=1,2,3,\cdot \cdot \cdot ,N$$ (32)

Where $x_{i}$ represents the position of the *i* food source;UBand LB are the upper and lower limits of the search space respectively;r is a random number between [0,1].

The food source access table is initialized via Equation (33).


$$S_{i,j}=\left\{ \begin{array}{c} 0,i\neq j\\ \text{null,i=j} \end{array}\right.\;,i=1,\cdot \cdot \cdot ,N,j=1,\cdot \cdot \cdot ,N$$ (33)

Where *i = j* indicates that hummingbirds forage for specific food sources; *i ≠ j* indicates that the current *j* food source was visited by the *i* hummingbird.

During flight, hummingbirds exhibit three flight modes: diagonal, axial, and omnidirectional. Each individual selects among these modes with equal probability. Since the flight process is not the main research content of this paper, its specific model will not be introduced in detail.

#### Guided foraging.

When rand < 0.5, hummingbirds conduct guided foraging according to their current flight skills, and obtain candidate food sources by visiting target food sources. The candidate food source location is updated as defined in Equation (34):


$$v_{m}(t+1)=x_{m,tar}(t)+a\times D\times [\begin{array}{c} x_{m}(t)-x_{m,tar}(t) \end{array}]$$ (34)

Where $v_{m}(t+1)$ represents the position of the m-th candidate food source in *t + 1* iteration;$x_{m}(t)$ represents the position of the m-th candidate food source in the t iteration;$x_{m,tar}(t)$ indicates the location where the m-th hummingbird will visit the target food source;a is a guiding foraging factor that follows the standard normal distribution.

The food source location is updated via Equation (35):


$$\begin{array}{c} x_{m}(t+1)=\left\{ \begin{array}{cc} \;0, & x_{m}(t)F\left(x_{m}(t)\right)\leq F\left(v_{m}(t+1)\right)\\ v_{m}(t+1), & \;F\left(x_{m}(t)\right)>F\left(v_{m}(t+1)\right) \end{array}\right.\; \end{array}$$ (35)

Where F(·) represents the fitness value of the objective function.

#### Regional feeding.

When rand≥0.5, hummingbirds do regional foraging in areas adjacent to the current food source, with location updates defined by Equation (36).


$$v_{m}(t+1)=x_{m}(t)+b\times D\times x_{m}(t)$$ (36)

Where b is the regional foraging factor that follows the standard normal distribution.

#### Migrate for food.

AHA defines the migration coefficient M. When the number of iterations *t* reaches the migration coefficient M, hummingbirds migrate for food and replace the existing food source with other new food sources with the worst nectar replenishment rate. The updated food source locations are defined by Equation (37).


$$x_{\text{wor}}(t+1)=LB+r\times (UB-LB)$$ (37)

Where $x_{\text{wor}}$ represents the location of the food source with the worst nectar replenishment rate in the population.

### 5.2 Multi-target artificial hummingbird algorithm

In the multi-objective optimization problem, one objective is optimized at the expense of other objectives. In multi-objective problems, it is impossible to obtain a set of solutions with the minimum of each objective, but only a set of Pareto solutions can be obtained. The screening mechanism of MOAHA is as follows:

1. MOAHA introduces an external archive to store a fixed number of non-dominated solutions;
2. The congestion distance method based on dynamic elimination is adopted to maintain the diversity of solutions in the whole optimization process;
3. Adopt the solution update mechanism based on non-dominant sorting to update the scheme.

### 5.3 Improved multi-objective artificial hummingbird algorithm

#### 5.3.1 Integrating logistic-sine chaotic mapping with elite reverse learning to initialize the population.

Most heuristic optimization algorithms are highly dependent on the initial population position. A randomly distributed initial population often leads to uneven distribution, which can adversely affect the algorithm’s accuracy. The AHA also uses a random population initialization, resulting in an uneven distribution of individuals and a lack of population diversity. To address this, chaotic mapping is frequently used to improve population initialization in meta-heuristic algorithms [31]. Among the various chaotic models, Tent and Logistic are the most commonly used. Xia et al. applied logistic-sine chaotic mapping to generate the initial population, enhancing both the uniformity of the initial population and the quality of the initial solutions [32]. In this paper, we propose a chaotic reverse learning strategy based on a combination of logistic-sine chaotic mapping and elite reverse learning to initialize the population. The mathematical expression of logistic-sine chaotic mapping is given by Equation (38).


$$x_{i+1}=\mu \times x_{i}\times \left(1-x_{i}\right)+(4-\mu )\times sin\left(\pi \times x_{i}\right)/4$$ (38)

Where μ is the chaos coefficient, which is set in this paper μ =0.5.

First, N D-dimensional initial solutions $x_{i,j}$(*i = 1,2,…,N;j = 1,2,…,D*) are generated by logistic-sine chaotic mapping. Secondly, the initial solutions are sorted, and the corresponding extreme point for each individual is selected as the elite individual $x_{i,j,e}$*=*($x_{i,1,e}$*,*$x_{i,2,e}$*,...,*$x_{i,D,e}$). The chaos elite reverse solution $\overset{―}{x_{i,j,e}}$*=*($\overset{―}{x_{i,1,e}}$*,*$\overset{―}{x_{i,2,e}}$*,...,*$\overset{―}{x_{i,D,e}}$) is then generated according to Equation (39). Where the dynamic boundary is set by Equation (40) to regulate the position points that cross the boundary:


$$\overset{―}{x_{i,j,e}}=k\times \left(\alpha_{j}+\beta_{j}\right)-x_{i,j,e}$$ (39)

$$\overset{―}{x_{i,j,e}}=rand\left(\alpha_{j},\beta_{j}\right),\overset{―}{x_{i,j,e}}<\alpha_{j},or\overset{―}{x_{i,j,e}}<\beta_{j}$$ (40)

In the formula, the elite reverse coefficients k∈(0,1), $\alpha_{j}$=min($x_{i,j,e}$) and $\beta_{j}$=max($x_{i,j,e}$)

Finally, all the initial solutions and elite reverse solutions generated by the chaotic mapping are combined and sorted, and the best N solutions are selected as the initial population.The combined strategy integrating Logistic-Sine chaotic mapping with elite opposition-based learning for population initialization effectively addresses the limitations of conventional stochastic initialization methods. This synergistic approach prevents uneven distribution of population individuals while enhancing spatial coverage characteristics, thus mitigating premature diversity loss that critically impacts evolutionary optimization algorithms. The dual mechanisms ensure sufficient initialization heterogeneity through chaotic ergodicity and directional guidance from elite exemplars, establishing a robust foundation for subsequent global exploration phases.

**Fig 4:**
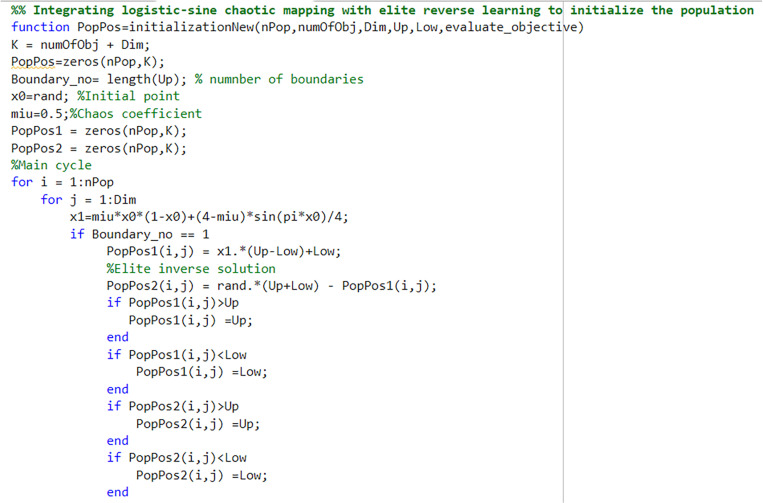
Integrating logistic-sine chaotic mapping with elite reverse learning to initialize the population.

**Fig 5:**
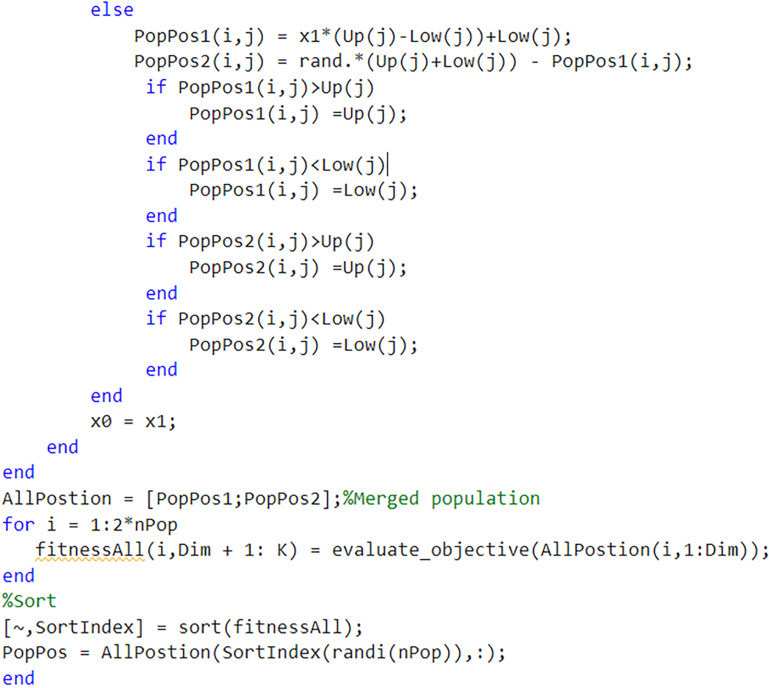
Integrating logistic-sine chaotic mapping with elite reverse learning to initialize the population.

Figs 4 and 5 illustrate the process of integrating logistic-sine chaotic mapping with elite reverse learning for population initialization.

#### 5.3.2 Adaptive spiral migration foraging.

In the AHA, migratory foraging behavior drives the least-fit hummingbird individuals to move randomly in search of better solutions. Inspired by the spiral search concept in the Whale Optimization Algorithm (WOA) [33], we propose an improvement to the migration-foraging behavior. The improved mathematical expression for migration and foraging is shown in Equation (41).


$$\begin{array}{c} \left\{ \begin{array}{c} x_{\text{wor}}(t+1)=\omega \times x_{\text{best}}(t)+\eta \times Abs\left(x_{\text{best}}(t)-x_{\text{wor}}(t)\right)\times e^{l}\times cos(2\pi l)\\ \omega =0.2\cos\left(\frac{\pi }{2}\times \left(1-\frac{t}{T_{\max}}\right)\right)\\ \;\eta =exp\left(5cos\left(\pi \times \left(1-\frac{t}{T_{\max}}\right)\right)\right) \end{array}\right.\; \end{array}$$ (41)

Where ω is the adaptive inertia weight; η is an adaptive spiral parameter; and l is a random number ranging from 0 to 1. $T_{\max}$ is the maximum number of iterations.

**Fig 6:**
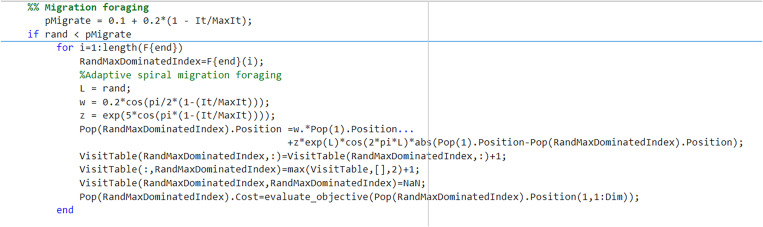
Adaptive spiral migration foraging.

At the beginning of the iteration, ω is smaller, meaning that the influence of the optimal hummingbird on the worst-positioned hummingbird is limited. At the same time, η is larger, which results in a larger spiral shape, allowing for a more thorough exploration of the solution space. In the later stages of the iterations, ω becomes larger, increasing the influence of the best hummingbirds on the worst hummingbirds. Concurrently, η decreases, reducing the size of the spiral and causing the worst-positioned hummingbird to move closer to the optimal position, thereby accelerating the convergence rate.In the adaptive spiral migration-foraging strategy, when relocating the hummingbird individual with the poorest fitness to a new position for stochastic search during the migration-foraging process, the algorithm initiates with a large spiral pattern to facilitate global exploration. Subsequently, it gradually reduces the spiral radius to enable local refinement. This dual-phase mechanism effectively guides the inferior individuals toward the optimal solution region while maintaining population diversity, thereby accelerating convergence rates through balanced exploration-exploitation dynamics. Fig 6 shows adaptive spiral migration foraging.

#### 5.3.3 Simplex method.

**Fig 7:**
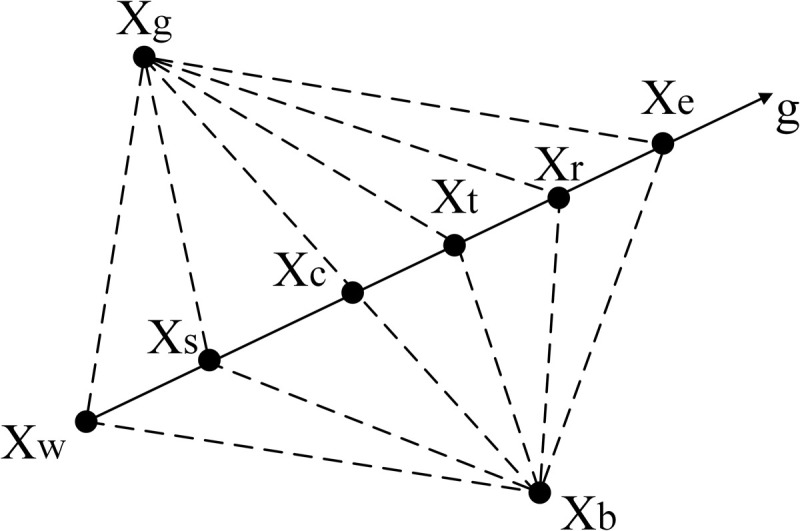
Simplex method.

The Simplex method is a polyhedral search algorithm known for its fast search speed and simple principles. It primarily determines whether the direction vector $X_{w}$ of the worst vertex *g* moves towards the best solution through iteration. The movement is controlled by reflectin*g*, expanding, contracting, and reducing the search space. The Simplex method is depicted in Fig 7.

The Simplex method is implemented to examine the best advantage $X_{g}$, the secondary advantage $X_{b}$, and the maximum disadvantage $X_{w}$, and subsequently calculate the position of the center point $X_{c}$.

A reflection operation is performed on the worst vertex $X_{w}$, as shown in Equation (42).


$$X_{r}=X_{c}+\alpha (X_{c}-X_{w})$$ (42)

Where α is the reflection coefficient, and α = 1.

If $X_{w}$>$X_{c}$, the reflection direction is correct. In this case, an expansion operation is performed, as shown in Equation (43).


$$X_{e}=X_{c}+\beta (X_{r}-X_{c})$$ (43)

Where β is the expansion coefficient, and β = 2.

If $X_{e}$>$X_{g}$, the expansion point $X_{e}$ replaces the maximum disadvantage point $X_{w}$. Otherwise, the reflection point $X_{r}$ replaces the maximum disadvantage point $X_{w}$.

If $X_{g}$>$X_{r}$ and the reflection direction is incorrect, an external contraction operation is performed, as shown in Equation (44).


$$X_{t}=X_{c}+\gamma (X_{w}-X_{c})$$ (44)

Where γ is the external contraction coefficient, and γ = 0.5.

If $X_{t}$>$X_{w}$, the external contraction point $X_{t}$ replaces the maximum disadvantage point $X_{w}$.

If $X_{w}$<$X_{r}$<$X_{g}$, an internal contraction operation is performed, as shown in Equation (45).


$$X_{s}=X_{c}-\mu (X_{w}-X_{c})$$ (45)

Where μ is the internal shrinkage coefficient, and μ = 0.5.

If $X_{s}$>$X_{w}$, the internal contraction point $X_{s}$ replaces the maximum disadvantage point $X_{w}$. Otherwise, the reflection point $X_{r}$ replaces the maximum disadvantage point $X_{w}$.

**Fig 8:**
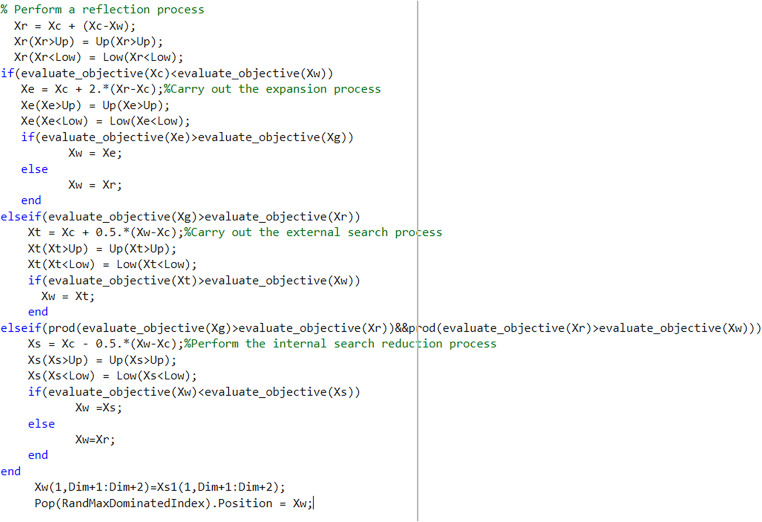
Simplex method.

By incorporating the Nelder-Mead simplex method into the iterative framework, this methodology systematically enhances population quality through three core operations. The reflection operation allows the worst-performing individual to explore all feasible solutions while avoiding premature convergence. Inner/outer contraction operations facilitate escaping from suboptimal positions while maintaining solution space coverage. Most critically, the expansion operation empowers elite solutions to escape local minima by conducting counter-directional searches away from the worst individual’s position. This systematic approach not only strengthens local search capability but also balances exploitation-exploration dynamics, ultimately improving global optimization performance. Fig 8 shows the procedure of the simplex method.

**Fig 9:**
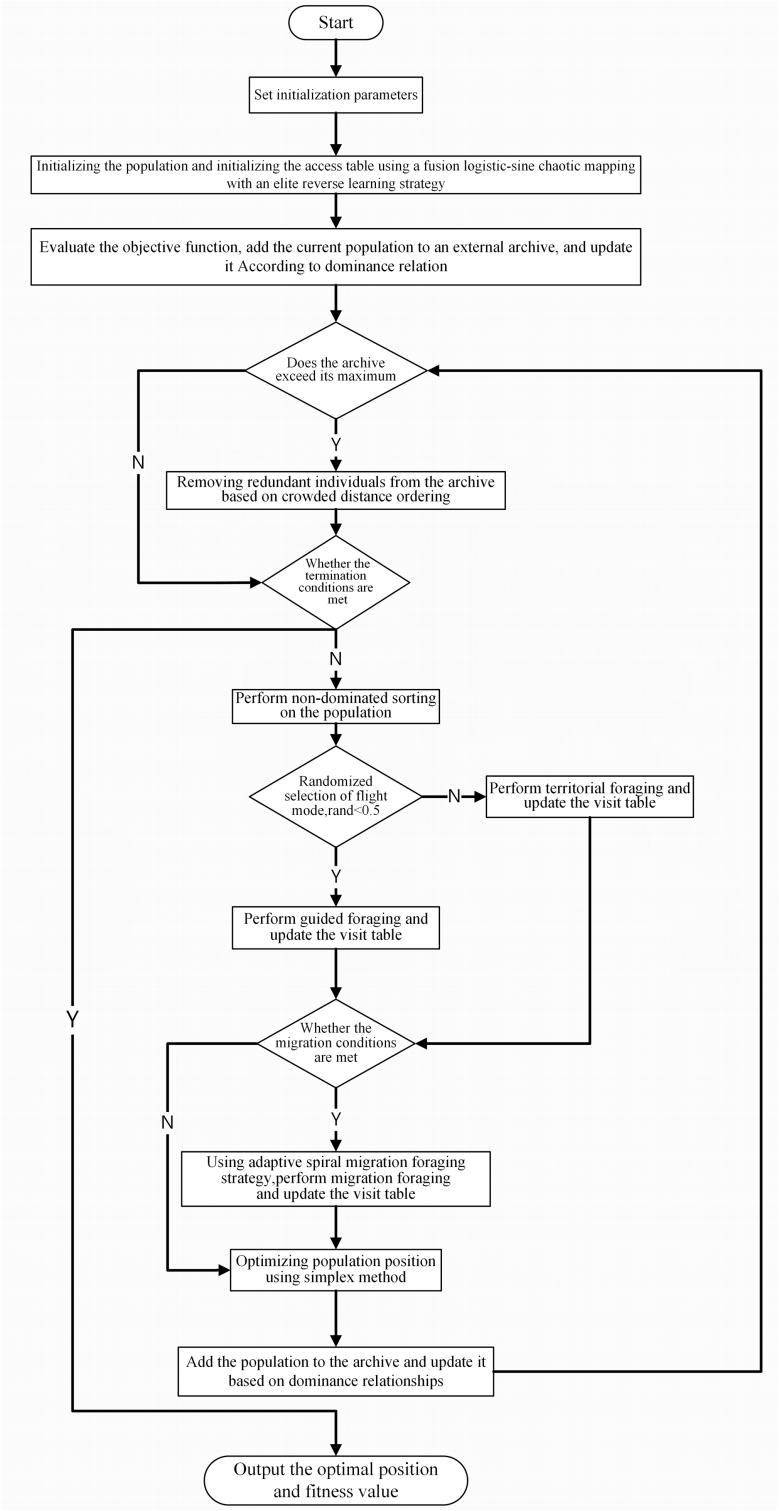
Flow chart of improved multi-objective artificial hummingbird algorithm.

The flowchart for the improved MOAHA is shown in Fig 9.

### 5.4 Algorithm performance verification

**Fig 10:**
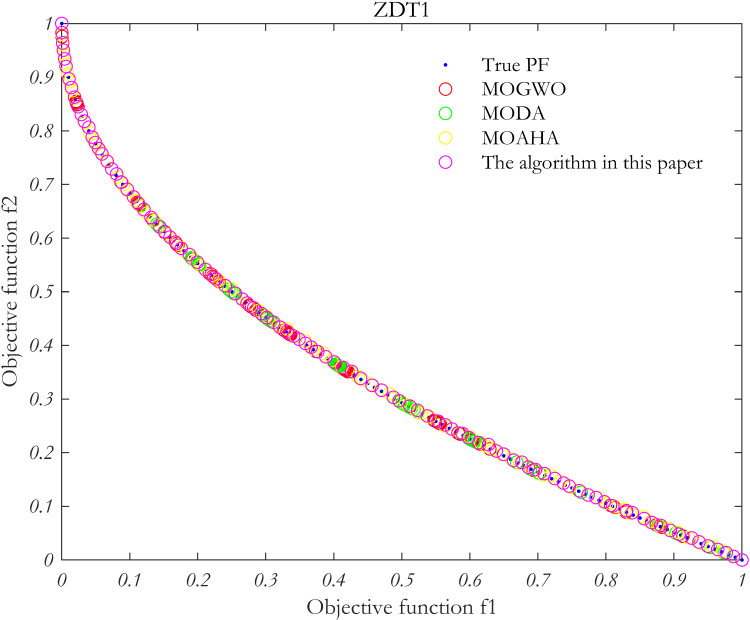
Simulation results of MOP standard test function ZDT1.

**Fig 11:**
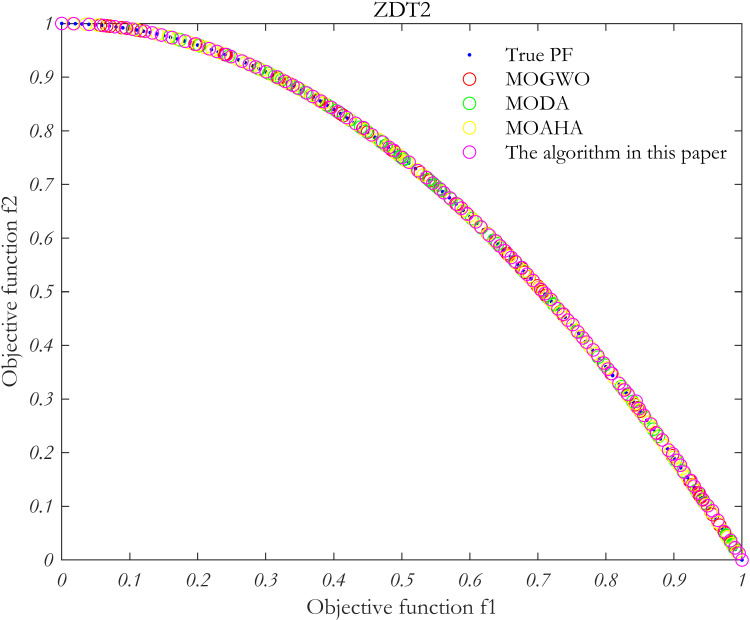
Simulation results of MOP standard test function ZDT2.

**Fig 12:**
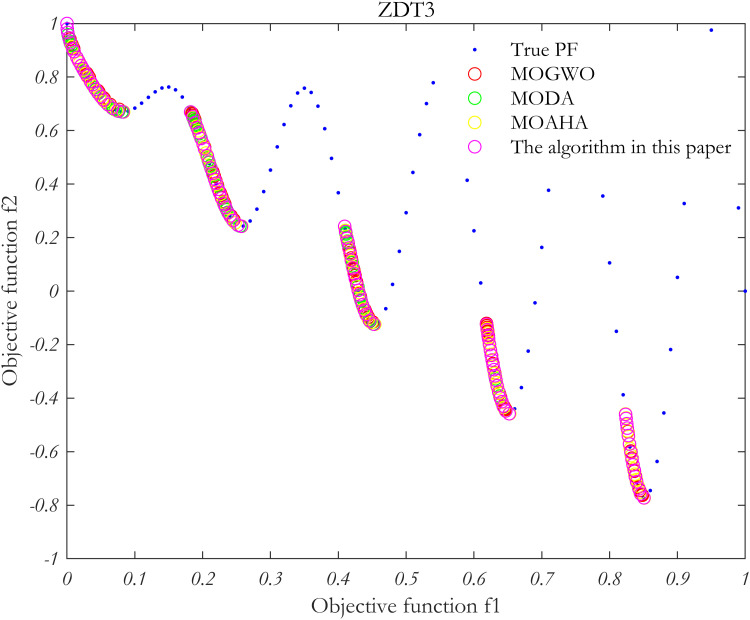
Simulation results of MOP standard test function ZDT3.

**Fig 13:**
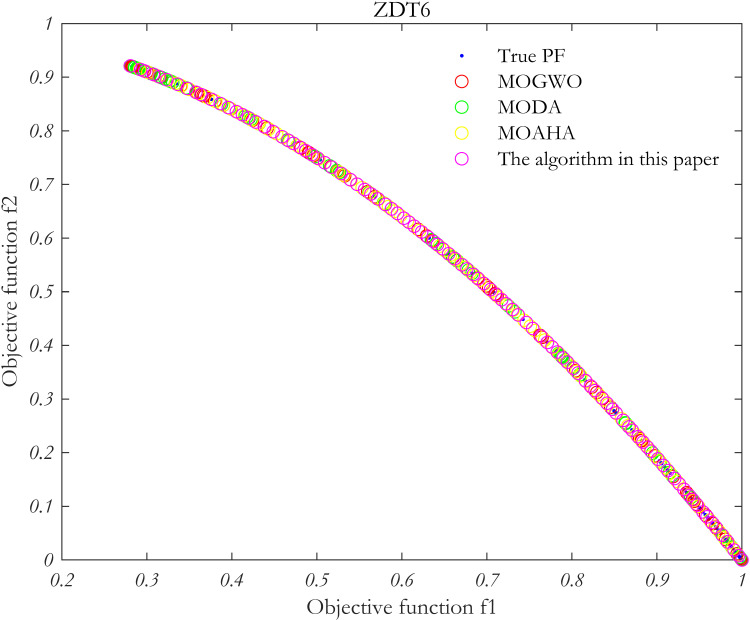
Simulation results of MOP standard test function ZDT6.

In order to verify the performance of the proposed algorithm, MOP test function is solved by using the proposed algorithm, MOAHA algorithm, MOGWO algorithm and MODA algorithm respectively. The parameters of the four algorithms are the same, the population size is 100, the number of iterations is 500, and the external archive is 100. Figs 10–13 show the simulation results.

The performance of the algorithm is evaluated using two metrics: the IGD index and the Spacing index. These metrics are used to analyze the Pareto frontiers obtained from the four multi-objective problem-solving methods discussed above.

The IGD index measures the distance between the non-dominated solution set produced by the algorithm and the actual Pareto frontier. It serves as an indicator of both the convergence and diversity of the algorithm’s performance. Specifically, the IGD index is calculated as the average Euclidean distance from each point on the true Pareto front to the closest point in the non-dominated set. A lower IGD value suggests that the generated solution set is more aligned with the true Pareto frontier. The mathematical expression for the IGD index is provided in Equation (46).


$$IGD(P)=\frac{\sum_{x\in P^{*}}mind(x,P)}{|P^{*}|}$$ (46)

Where *P* is the Pareto solution set obtained by the algorithm; $P^{*}$ is the optimal solution set of the multi-objective problem; mind(.) is the symbol for calculating the average value.

The Spacing index evaluates the average distance between solutions within the non-dominated set generated by the algorithm, providing insight into the solution distribution. This index is determined by calculating the standard deviation of the minimum distances from each solution to the others in the set. A smaller Spacing value indicates that the solutions are more closely packed and more uniformly distributed. The formula for calculating the Spacing index is given by Equation (47).


$$Spacing(P)=\left(\frac{\sum_{i=1}^{|P|}{\left(\overset{―}{d}-d_{i}\right)}^{2}}{|P-1|}\right)$$ (47)

Where $\overset{―}{d}$ is the average of all $d_{i}$; $d_{i}$ denotes the shortest distance from the ith solution to all other solutions in the solution space.

The performance evaluation metrics for the four algorithms were calculated, and the results are shown in Table 2.

**Table 2:** Algorithm performance evaluation index results.

Test function	Index	MOGWO	MODA	MOAHA	The algorithm in this paper
ZDT1	IGD	0.012984	0.024413	0.0024394	0.0023126
	Spacing	0.01317	0.26559	0.0037197	0.0033773
ZDT2	IGD	0.0058498	0.0050581	0.0025225	0.0024034
	Spacing	0.0079424	0.0064633	0.0036172	0.0034111
ZDT3	IGD	0.012518	0.027773	0.0048794	0.0046054
	Spacing	0.012763	0.010672	0.0064069	0.0060585
ZDT6	IGD	0.0034893	0.0040693	0.0021258	0.0019509
	Spacing	0.011178	0.12212	0.0019194	0.0017878

As summarized in Table 2, in the multi-objective optimization problem represented by the four test functions, the IGD and Spacing indices for the proposed algorithm (MOAHA) are significantly better than those for the other three algorithms. This indicates that the solution set obtained by the MOAHA is not only closer to the real Pareto frontier but also more evenly distributed and diverse compared to the solutions obtained by the other algorithms. In conclusion, the results demonstrate that the improved MOAHA proposed in this paper is highly effective in solving multi-objective optimization problems.

## 6. Simulation example analysis

**Fig 14:**
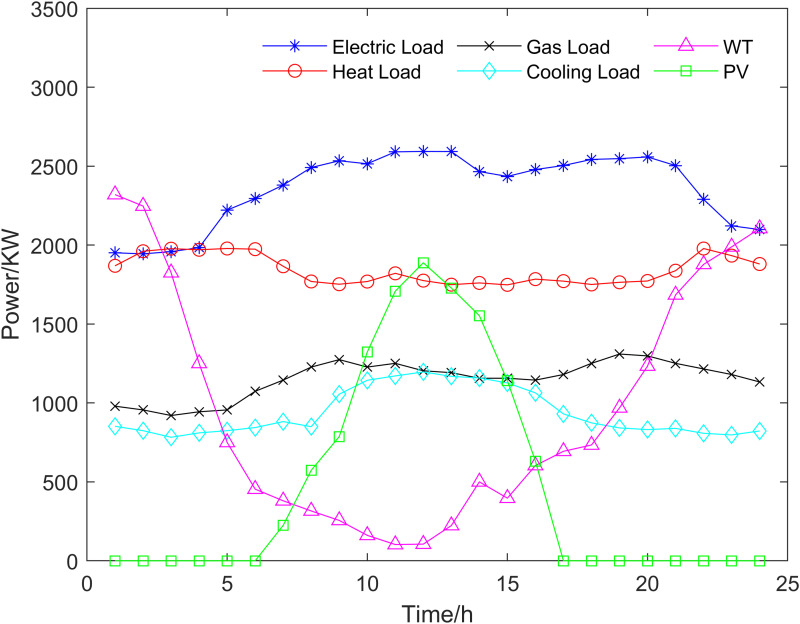
Electricity, heat, cold, gas load and new energy output.

In order to verify the effectiveness of the proposed algorithm in solving the multi-objective optimization problem of the integrated energy system, the integrated energy system of an industrial park in North China is taken as an example for simulation analysis. Electricity price peak period (11:00–14:00,18:00–21:00) is 0.83 yuan/KWH; The normal period (7:00–10:00,15:00–17:00) is 0.51 yuan/KWH; The valley time (1:00–6:00,22:00–24:00) is 0.3 yuan/KWH; The price of natural gas is 3.5 yuan/m3. The load of electricity, heat, cold and gas and the output of new energy are shown in Fig 14. The parameters of each unit in the system are shown in Table 3.

**Table 3:** System parameters of each unit.

Argument	Numerical value	Argument	Numerical value
$P_{CCHP}^{\max}$	3MW	$C_{AC}^{\max}$	2.5MW
$R_{WHB}^{\max}$	3MW	$C_{ER}^{\max}$	1.5MW
$P_{CCS,e}^{\max}$	2MW	$P_{EL,e}^{\max}$	2MW
$P_{MR}^{\max}$	0.8MW	$R_{EB}^{\max}$	1.5MW
$P_{HFC}^{\max}$	0.8MW	$\eta_{e}$	0.6
$\eta_{loss}$	0.05	$\eta_{h}$	1.9
$\eta_{H}$	0.95	$r^{c}$	0.4t/MW•h
$\eta_{c}$	2.4	$\lambda^{c}$	0.392t/MW•h
$r^{e}$	1.08t/MW•h	α	250¥/t
$\lambda^{e}$	0.5t/MW•h	g	0.39
l	2t	$\eta_{CCS}$	0.85
$\lambda_{CCS}$	0.31MW•h/t	$\eta_{HFC,h}$	0.9
$\eta_{EL}$	0.88	$\eta_{HFC,e}$	0.9
$\eta_{MR}$	0.55	$R_{{CH}_{4}}$	36MJ/m^3^
$\rho_{{\text{CO}}_{2}}$	2 kg/m^3^	$R_{EH}$	3600MJ/MW
$\eta_{EB}$	0.9	$\eta_{ER}$	2
$P_{\text{ES,e}}^{\text{min}}$	0MW	$P_{\text{ES,e}}^{\text{max}}$	1MW
$P_{\text{ES,H}}^{\text{min}}$	0m^3^	$P_{\text{ES,H}}^{\text{max}}$	500m^3^

### 6.1 Model optimization and solution

**Fig 15:**
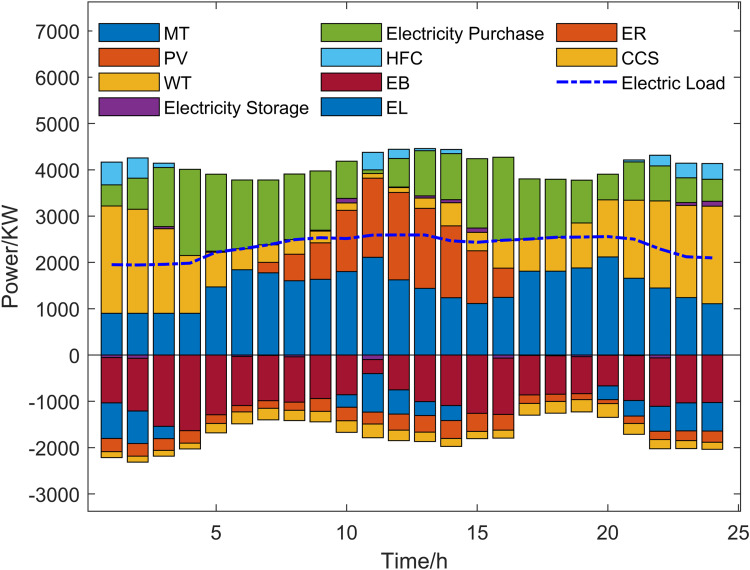
Electric power optimization results.

**Fig 16:**
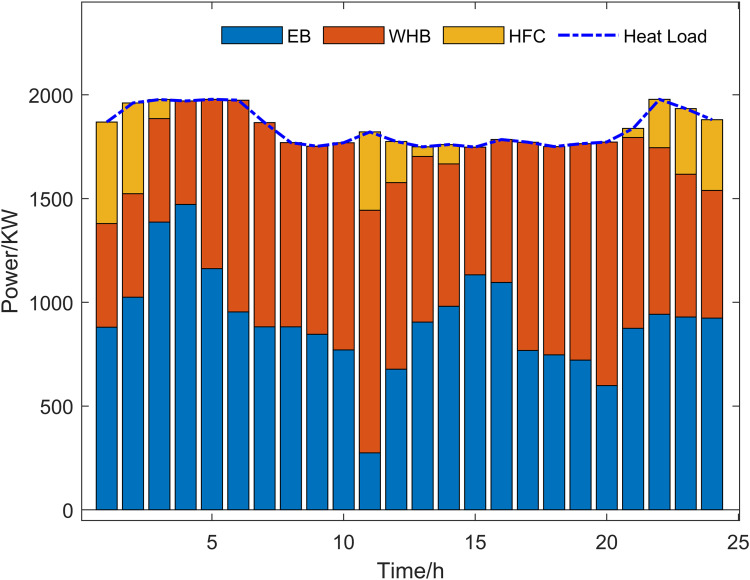
Thermal power optimization results.

**Fig 17:**
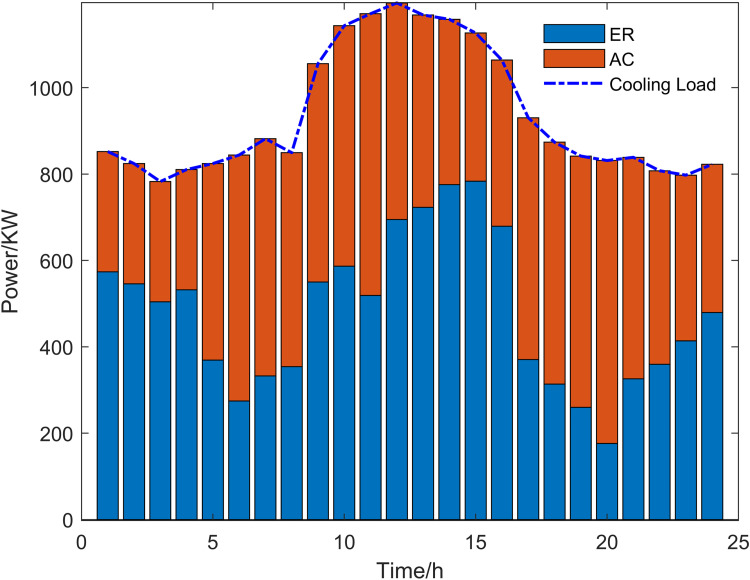
Cold power optimization results.

**Fig 18:**
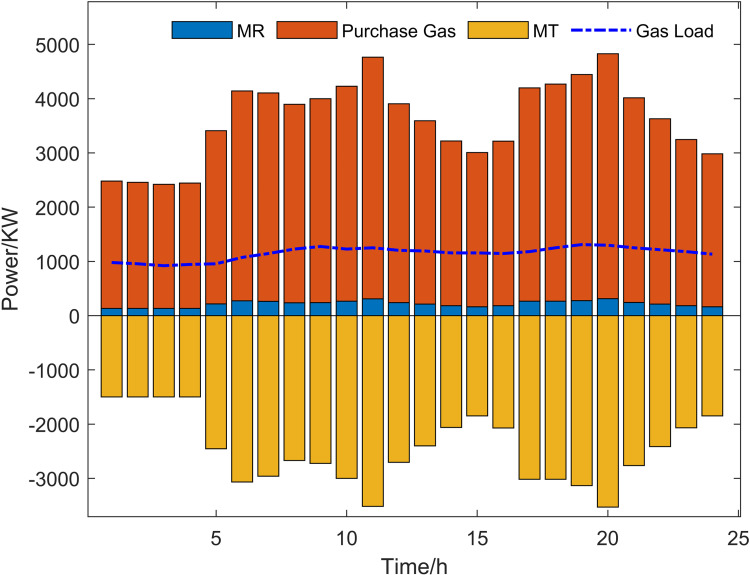
Gas power optimization results.

**Fig 19:**
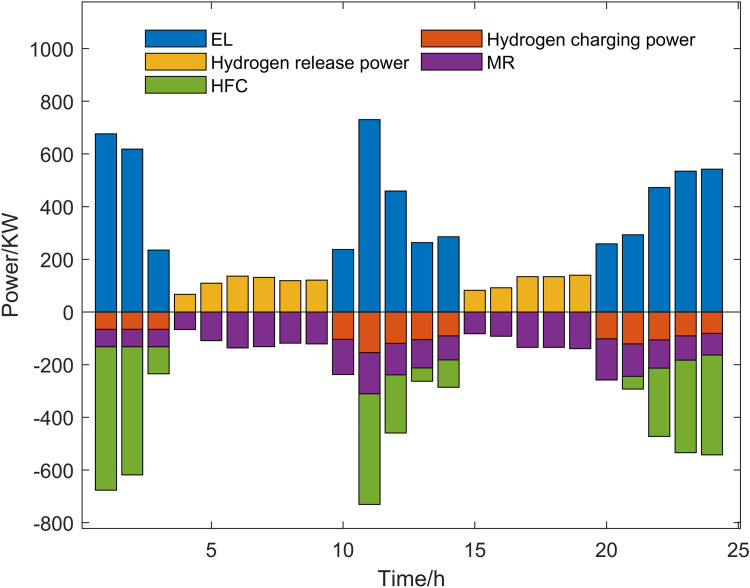
Hydrogen power optimization results.

**Fig 20:**
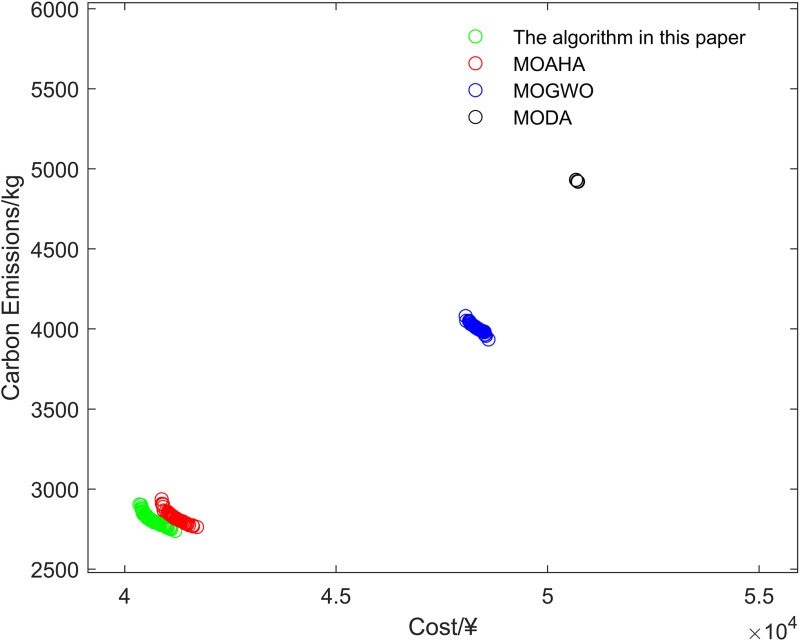
Four Pareto frontiers of algorithms.

To evaluate the performance of the proposed model in terms of carbon emission reduction and cost efficiency, three distinct scenarios are analyzed:

Scenario 1: The model does not consider the carbon capture device but includes P2G equipment and the stepped carbon trading mechanism;

Scenario 2: The model does not consider the carbon capture device but adopts the refined two-stage P2G operation and the stepped carbon trading mechanism;

Scenario 3: This model incorporates the carbon capture device, refined two-stage P2G operation, and the stepped carbon trading mechanism, representing the optimization model proposed in this study;

Scenario 3 represents the proposed optimization model, with its optimized operational outcomes illustrated in Figs 15–19 and solved using the improved multi-objective artificial hummingbird algorithm for case studies under a 24-hour operational cycle, where comparative analysis of four algorithms yields the Pareto front shown in Fig 20, configured with a population size of 100, 500 iterations, and an external archive capacity of 50.

### 6.2 Comparative analysis of different scenarios

Fig 20 depicts the Pareto frontiers of the proposed integrated energy system model generated by four comparative algorithms. Simulation results indicate that the developed methodology achieves superior Pareto-optimal solutions in multi-objective scheduling optimization for integrated energy systems, outperforming benchmark approaches in simultaneously balancing economic efficiency and carbon emission mitigation.

The simulation results for the three scenarios are presented in Table 4.

**Table 4:** Results of different scenarios.

Argument	Scenario 1	Scenario 2	Scenario 3
Cost of purchasing power/yuan	40389.55	38606.47	35661.78
Operation and maintenance cost/yuan	3737.66	4627.01	4876.42
Carbon emissions per kg	16481.71	13497.90	2817.63
Carbon transaction cost/yuan	5805.64	4686.71	755.51
Comprehensive cost/yuan	49932.85	47920.19	41293.71

Compared to Scenario 1, where the two-stage P2G operation process is absent, the addition of this process in Scenario 2 (replacing the basic P2G equipment) results in a 19.22% increase in the operation and maintenance costs. However, by generating H_2_ through the electrolyzer, part of which is supplied to the HFC for electricity and heat generation, and part of which is used by the MR to produce natural gas, the system reduces overall energy loss. This leads to a 4.03% reduction in the comprehensive system cost, demonstrating that incorporating the detailed two-stage P2G operation process improves the system’s economic efficiency.

In the context of an Integrated Energy System, the introduction of carbon capture equipment in Scenario 3, compared to Scenario 2, led to a 5.11% increase in operation and maintenance costs. However, this addition resulted in a significant reduction of 10,680.27 kg in carbon emissions, corresponding to a 79.13% decrease. Moreover, by utilizing the captured CO₂ as a feedstock for the methanation process, the overall system cost was reduced by 13.83%. This demonstrates that incorporating carbon capture equipment into an IES can yield substantial economic and environmental benefits.

## 7. Conclusion

This paper develops a low-carbon economic optimization scheduling model for IES, integrating a carbon capture unit, a refined two-stage P2G process, and a stepped carbon trading mechanism. The model is optimized using the improved MOAHA, and the performance of three distinct scenarios is evaluated. The main conclusions are as follows:

The synergistic integration of power-to-gas facilities, combined cooling-heating-power systems, and advanced energy storage devices establishes a multi-energy complementarity framework that significantly enhances energy utilization efficiency. This configuration minimizes energy cascade losses through coordinated multi-carrier energy conversion while enabling closed-loop carbon recycling via methanation processes. Thus, the IES low-carbon economic operation is improved.

The integration of carbon capture facilities demonstrates significant economic and environmental benefits, achieving a system energy procurement cost reduction of 2,944.69 yuan (7.63% decrease rate) while simultaneously reducing carbon emissions by 10,680.27 kg, which consequently lowers carbon trading costs by 3,931.2 yuan.It shows that CCS equipment can better achieve the effect of reducing carbon emissions, so as to realize the low-carbon economic operation of IES.

By incorporating carbon capture equipment and refining the two-stage operation process of Power-to-Gas (P2G), the carbon emissions were reduced by 13,664.08 kg compared to the scenario without these considerations. Additionally, the carbon trading costs were lowered by 5,050.13 yuan. As a result, the overall integrated cost was decreased by 8,639.14 yuan, representing a reduction of 17.3%.The refined two-phase power-to-gas operation mechanism, serving as an advanced alternative to conventional P2G systems, demonstrates enhanced performance in renewable energy accommodation while achieving significant reductions in operational costs and carbon emissions. This optimized approach utilizes CO₂ captured through carbon capture and storage technology as the primary carbon source for methanation processes, thus establishing a closed-loop carbon cycle. Furthermore, surplus hydrogen output can be effectively channeled to hydrogen fuel cells, with the subsequent electrical and thermal energy generated from HFC operation supplying a portion of the energy demand for integrated energy systems. This synergistic configuration establishes an energy cascade utilization framework that substantially improves hydrogen energy conversion efficiency across multiple operational phases.

The enhanced multi-objective artificial hummingbird algorithm demonstrates superior capability in generating high-quality Pareto-optimal solutions for low-carbon economic dispatch problems in IES.This improved computational framework exhibits significant advantages in achieving optimal equilibrium between economic efficiency and environmental sustainability. The algorithm’s adaptive dynamic search strategy enables comprehensive exploration of the solution space while maintaining computational efficiency, thereby providing decision-makers with diversified optimal operational schemes that satisfy multiple conflicting objectives in IES management.

## Supporting information

S1 Fig All the pictures in the article. (ZIP)

S1 Table All the tables in the article. (DOCX)

S1 Data Original data. (XLSX)
