# Supplementary figures and images for: Research on optimal scheduling of integrated energy system based on improved multi-objective artificial hummingbird algorithm
Source: PLoS One. 2025 Jun 4;20(6):e0325310. doi: 10.1371/journal.pone.0325310 (PMC12136360; doi:10.1371/journal.pone.0325310)

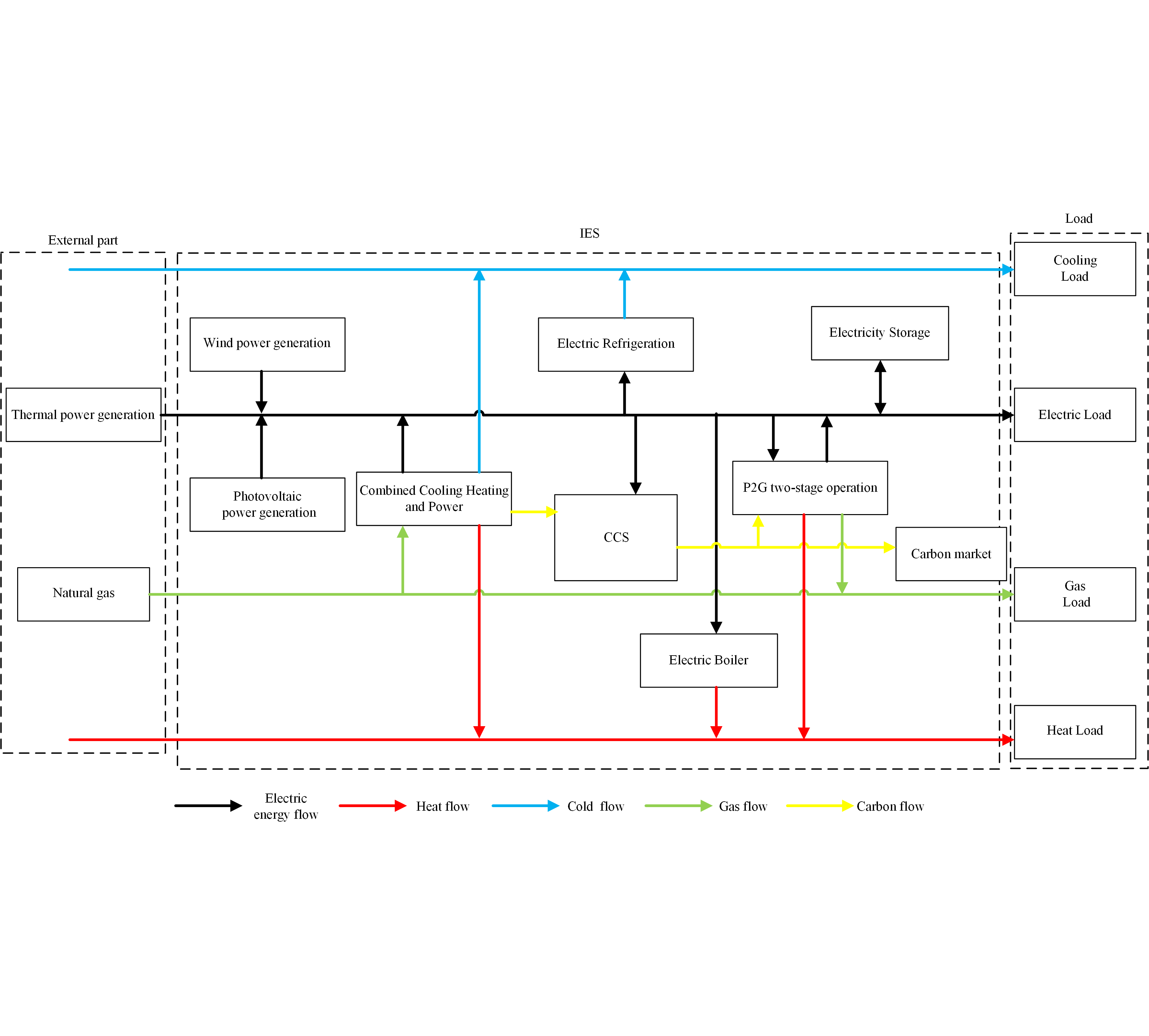

Supplement: S1 Fig — (ZIP) [file pone.0325310.s001.zip › S1 Fig/Fig 1.tif]

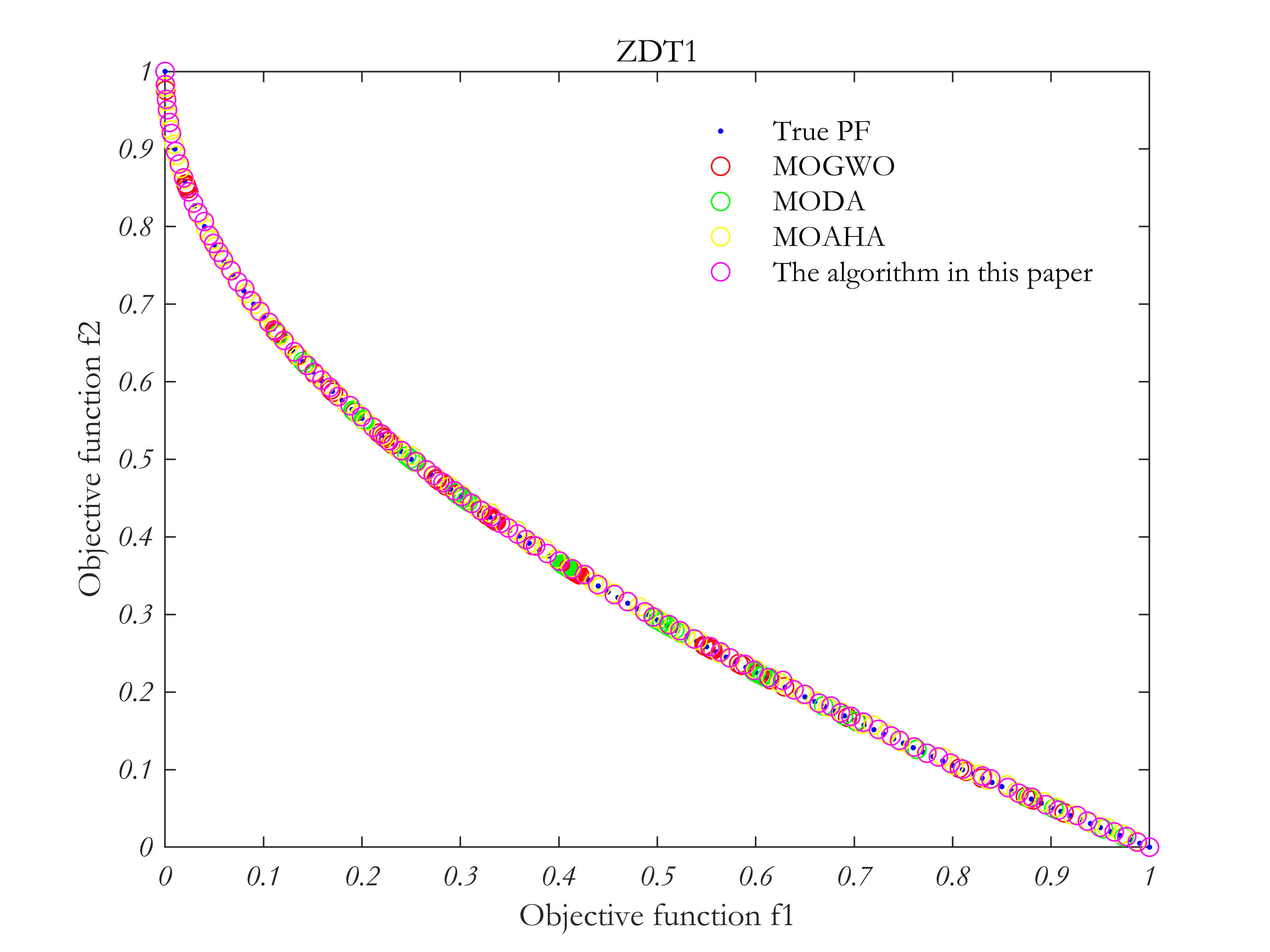

Supplement: S1 Fig — (ZIP) [file pone.0325310.s001.zip › S1 Fig/Fig 10.tif]

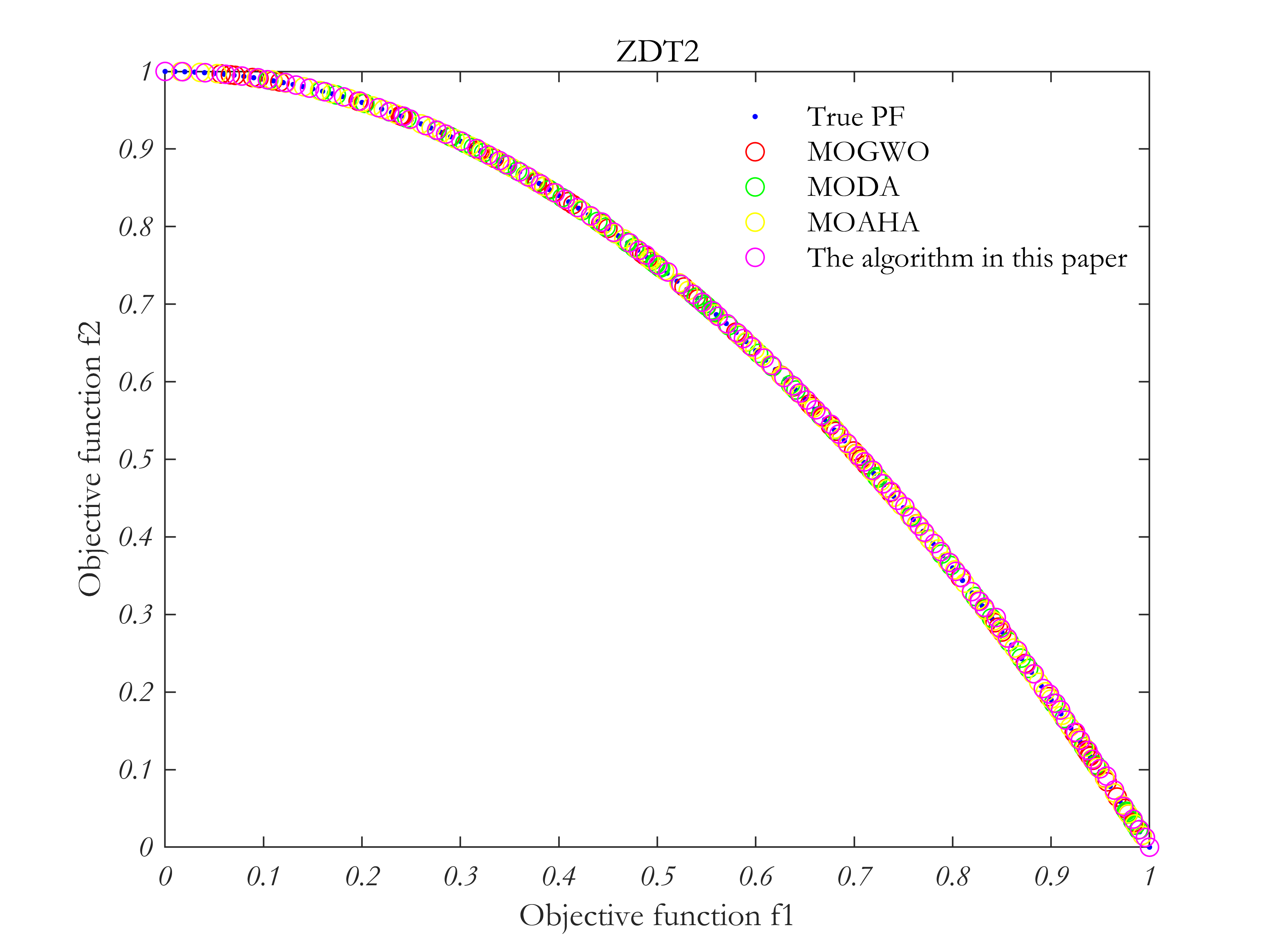

Supplement: S1 Fig — (ZIP) [file pone.0325310.s001.zip › S1 Fig/Fig 11.tif]

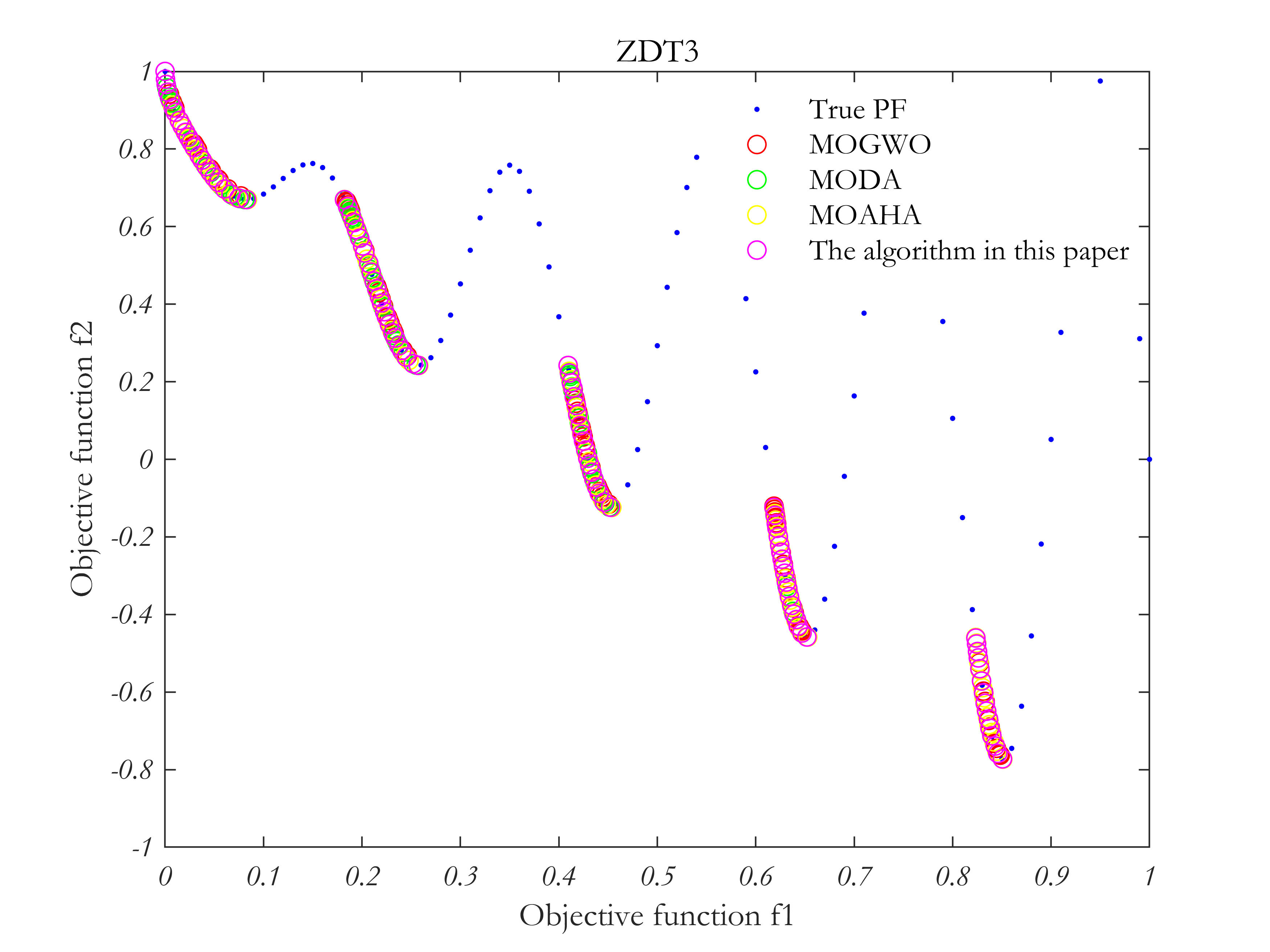

Supplement: S1 Fig — (ZIP) [file pone.0325310.s001.zip › S1 Fig/Fig 12.tif]

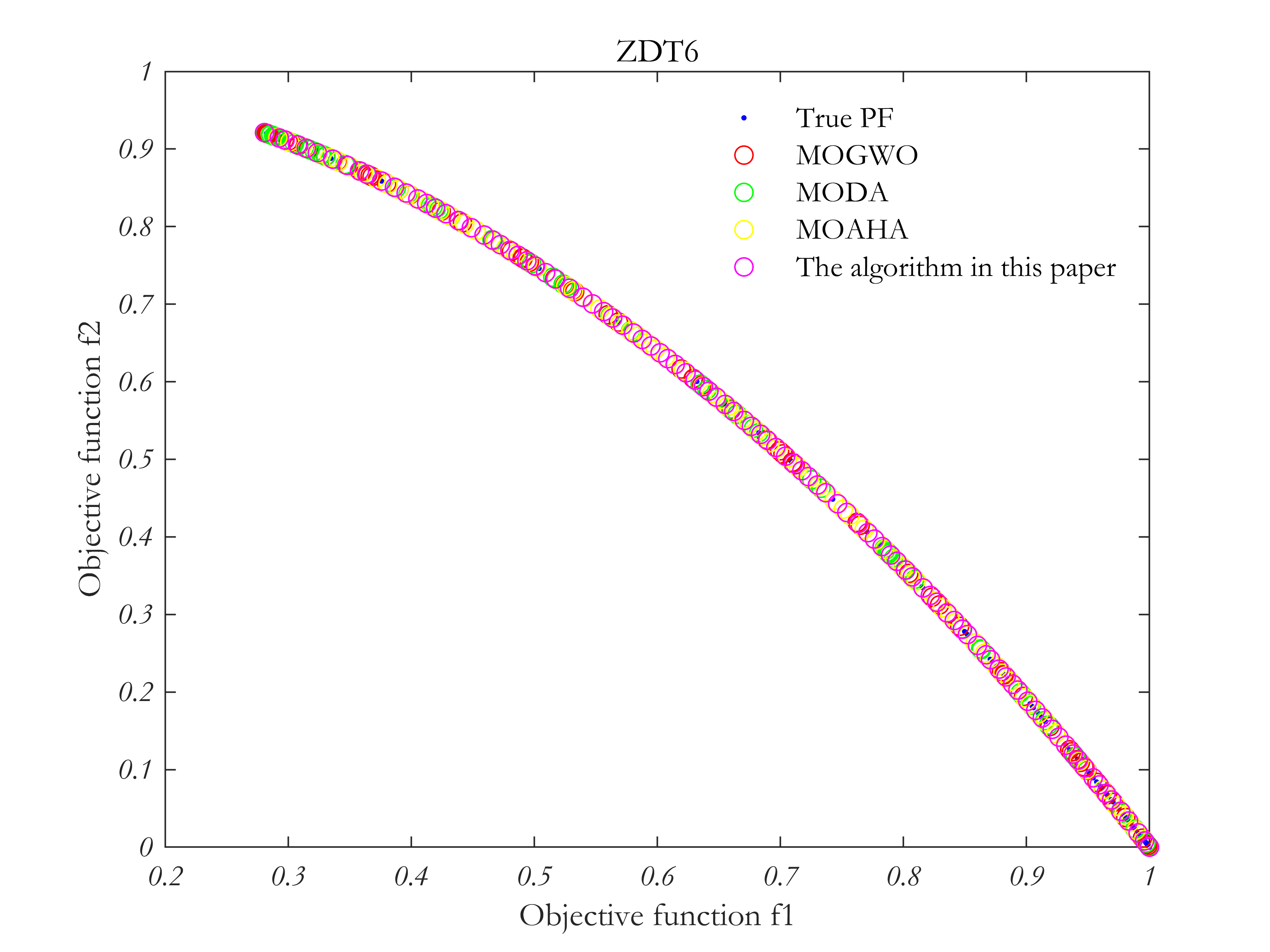

Supplement: S1 Fig — (ZIP) [file pone.0325310.s001.zip › S1 Fig/Fig 13.tif]

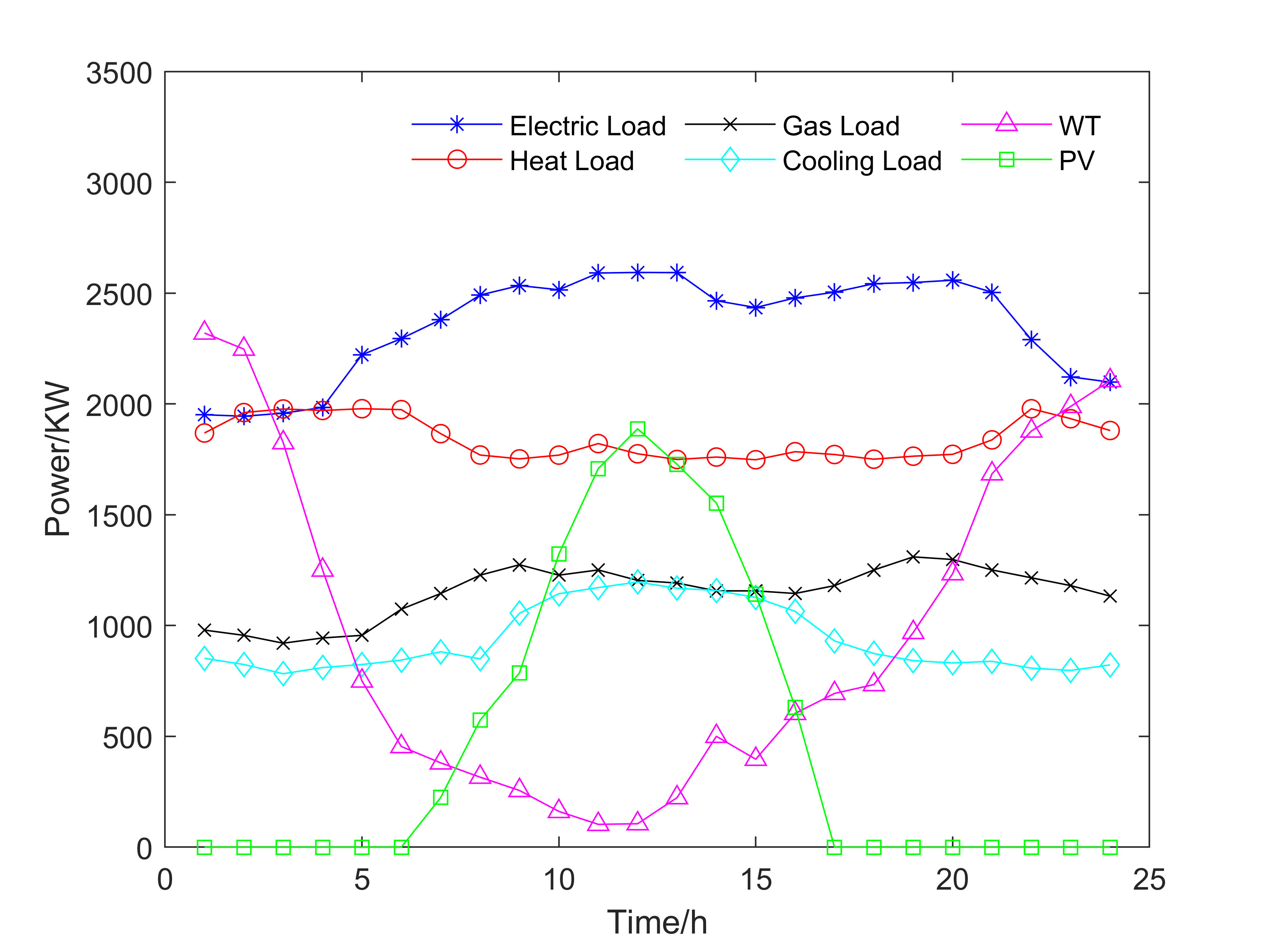

Supplement: S1 Fig — (ZIP) [file pone.0325310.s001.zip › S1 Fig/Fig 14.tif]

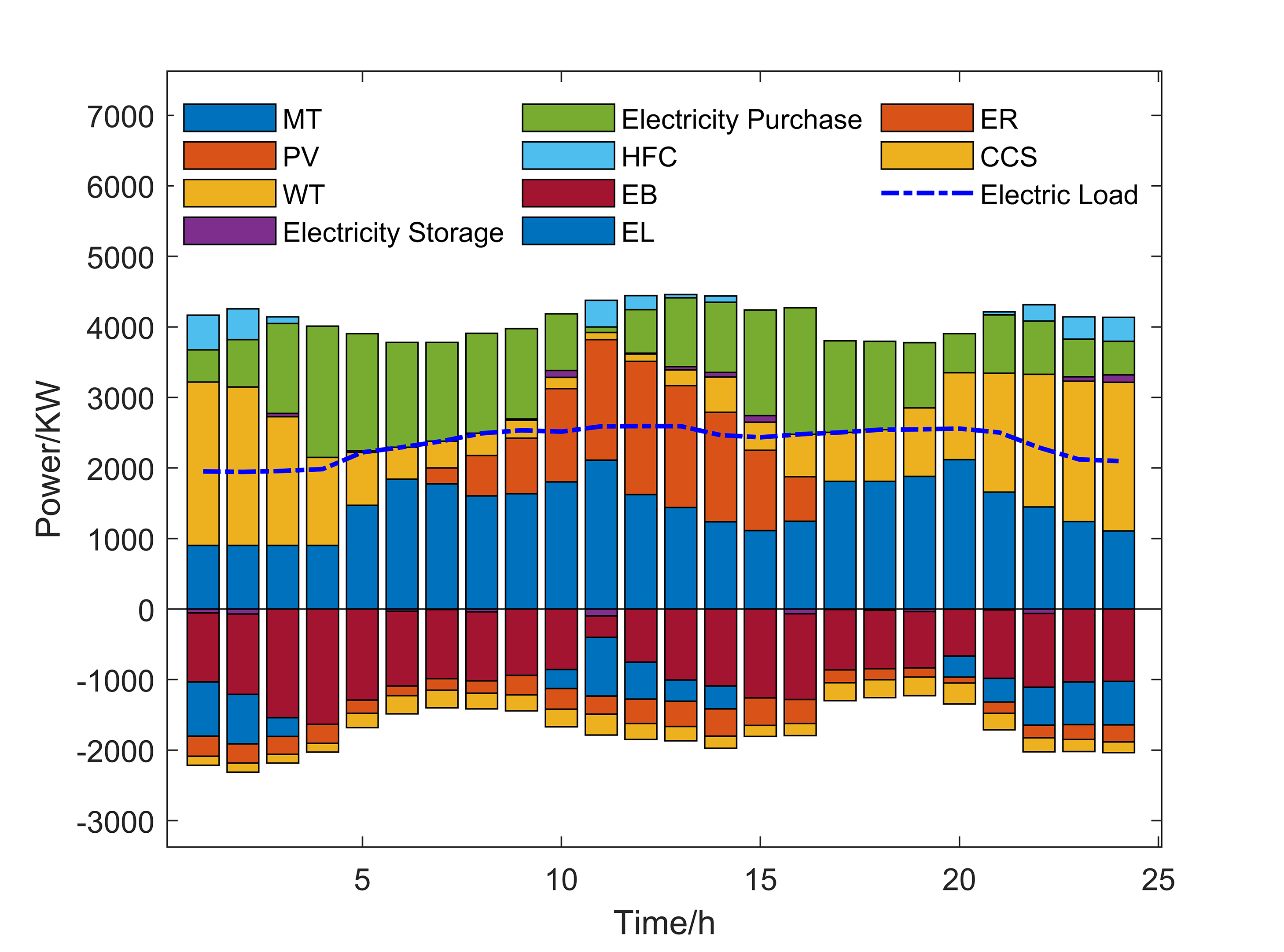

Supplement: S1 Fig — (ZIP) [file pone.0325310.s001.zip › S1 Fig/Fig 15.tif]

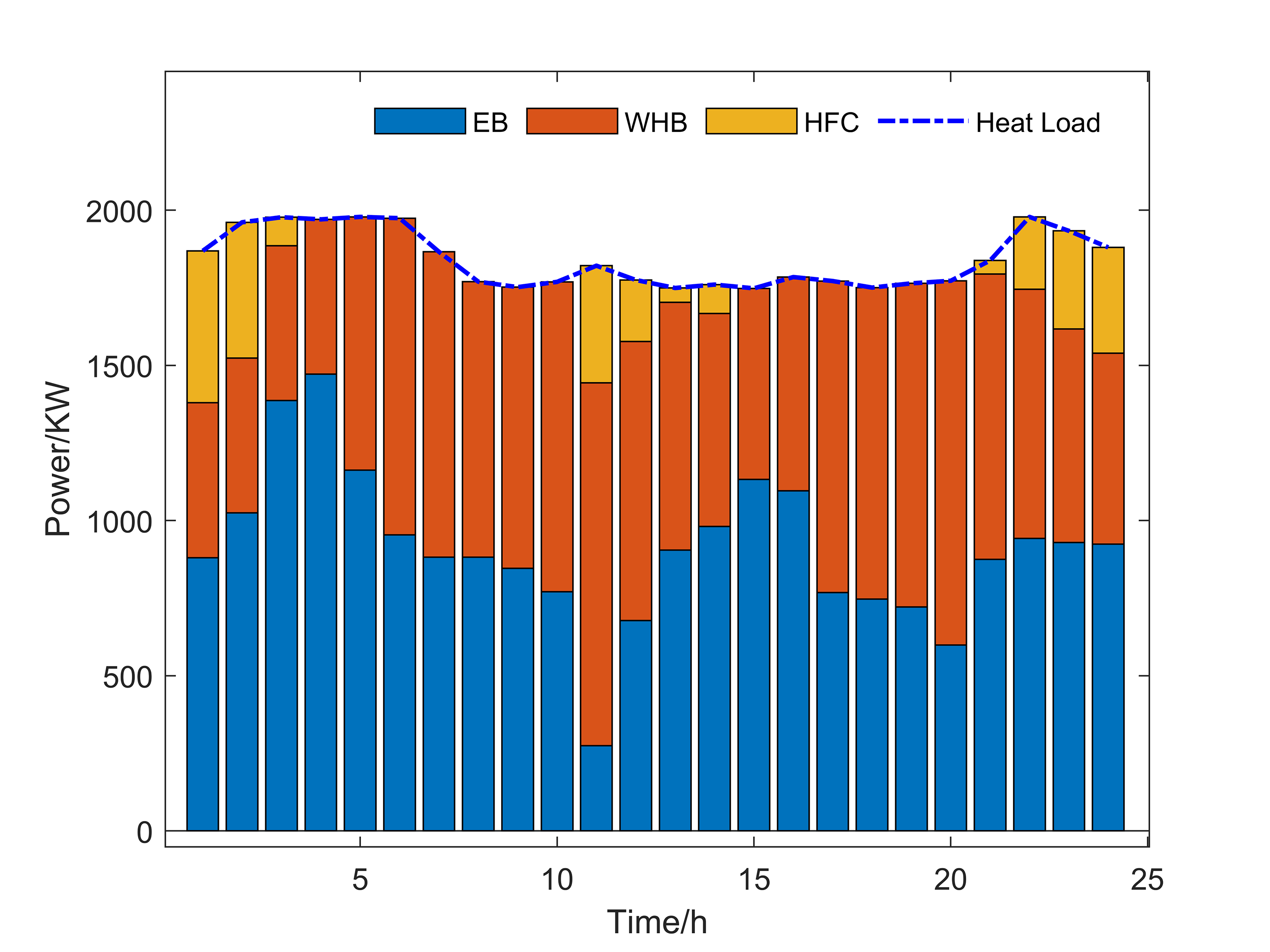

Supplement: S1 Fig — (ZIP) [file pone.0325310.s001.zip › S1 Fig/Fig 16.tif]

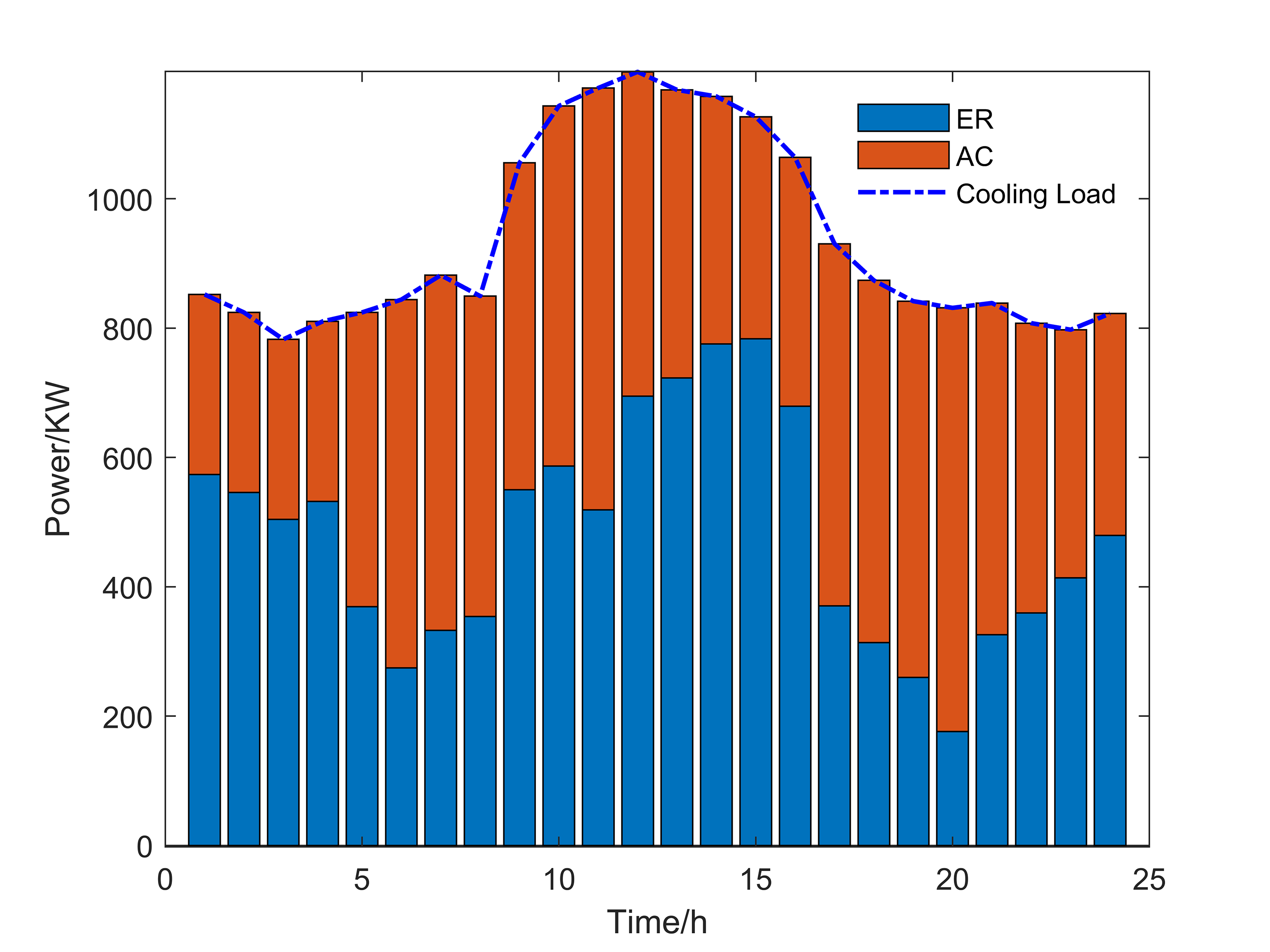

Supplement: S1 Fig — (ZIP) [file pone.0325310.s001.zip › S1 Fig/Fig 17.tif]

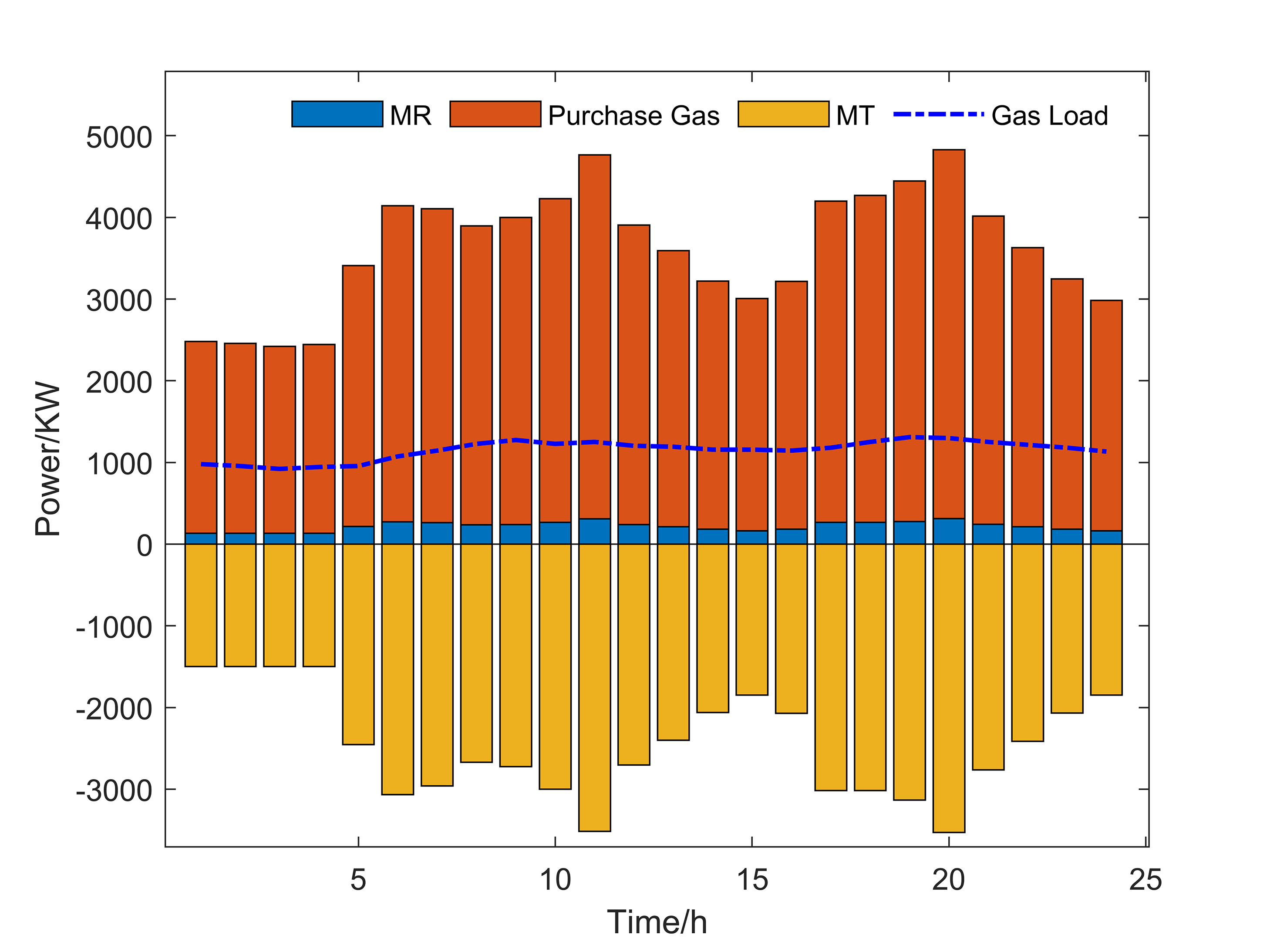

Supplement: S1 Fig — (ZIP) [file pone.0325310.s001.zip › S1 Fig/Fig 18.tif]

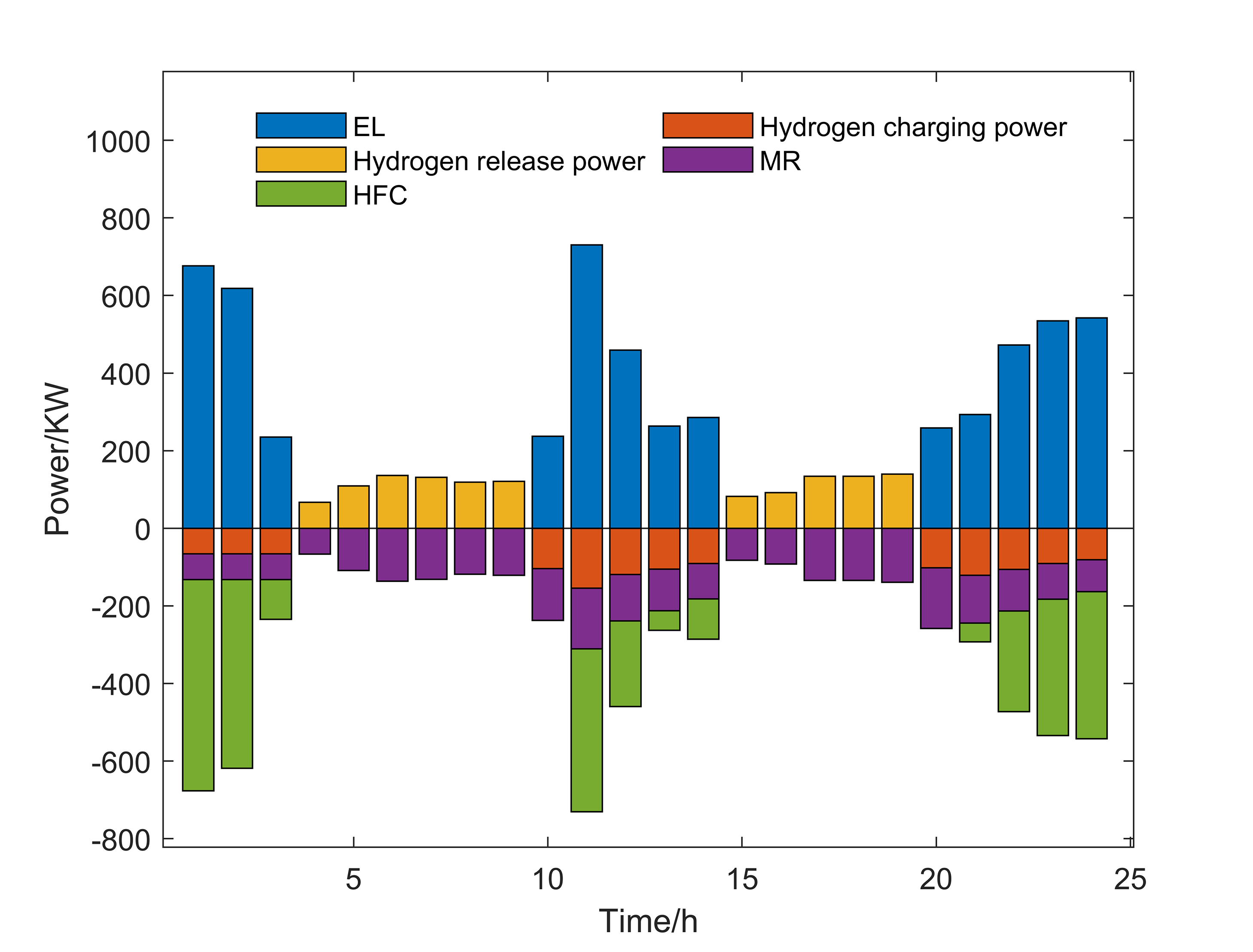

Supplement: S1 Fig — (ZIP) [file pone.0325310.s001.zip › S1 Fig/Fig 19.tif]

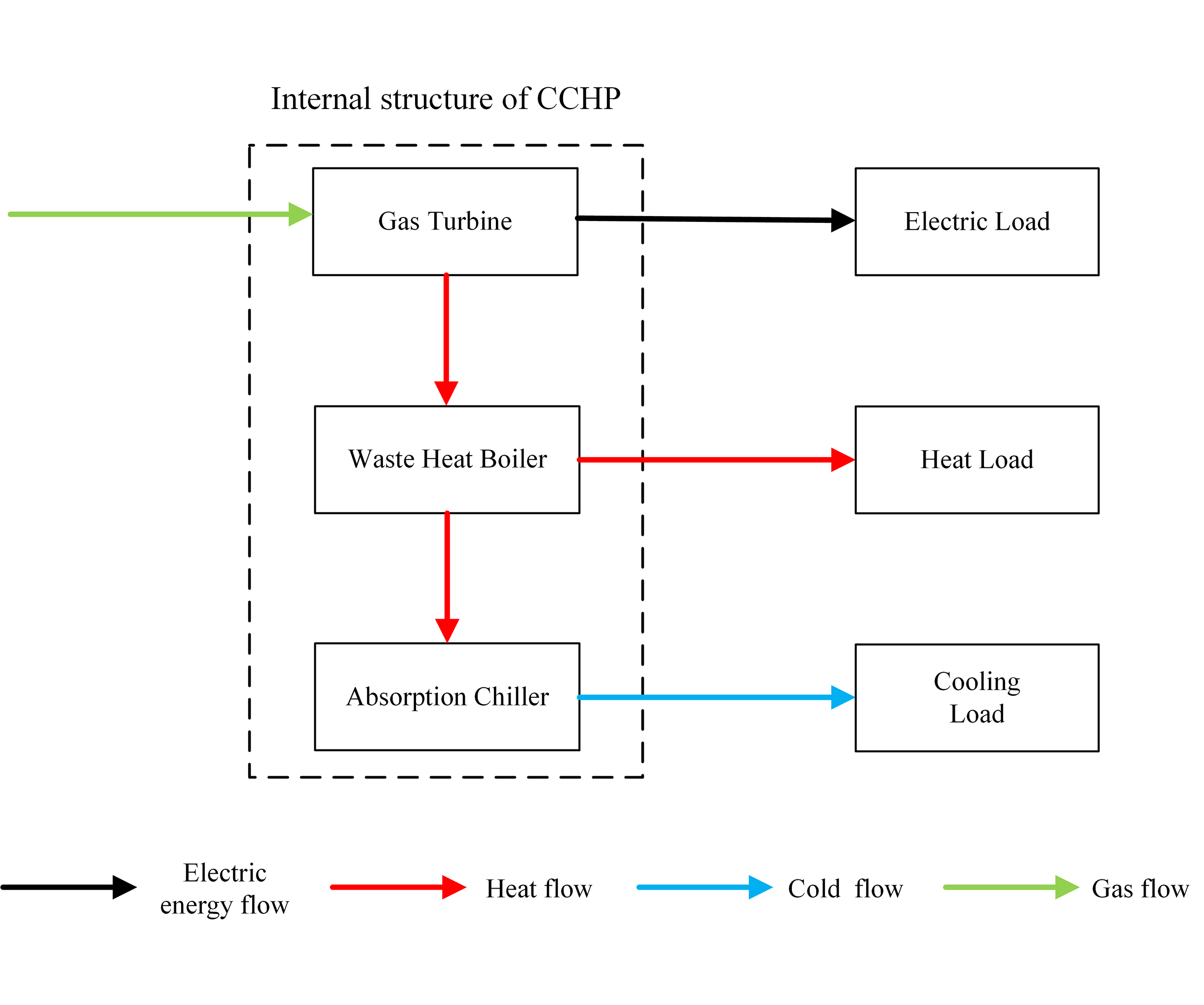

Supplement: S1 Fig — (ZIP) [file pone.0325310.s001.zip › S1 Fig/Fig 2.tif]

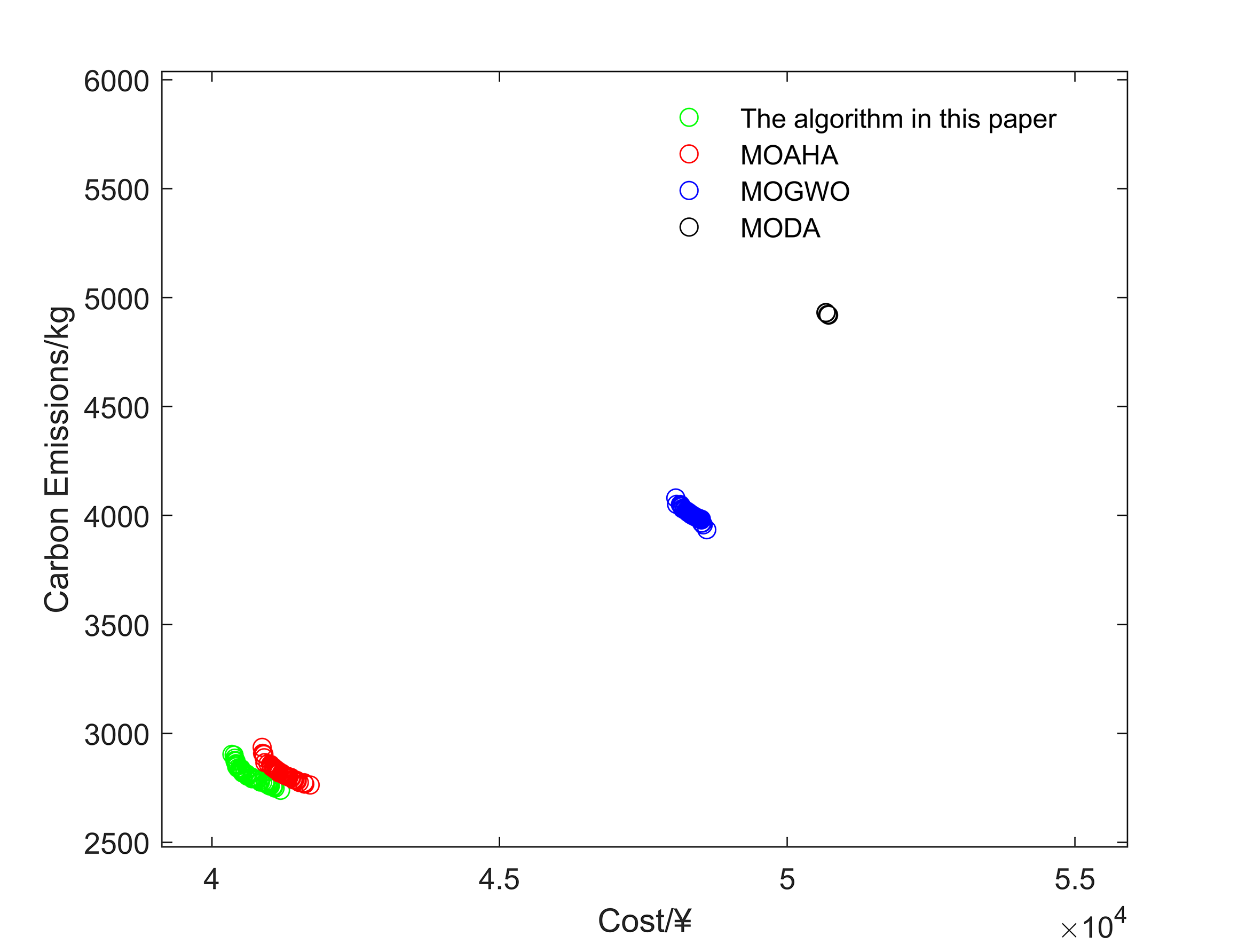

Supplement: S1 Fig — (ZIP) [file pone.0325310.s001.zip › S1 Fig/Fig 20.tif]

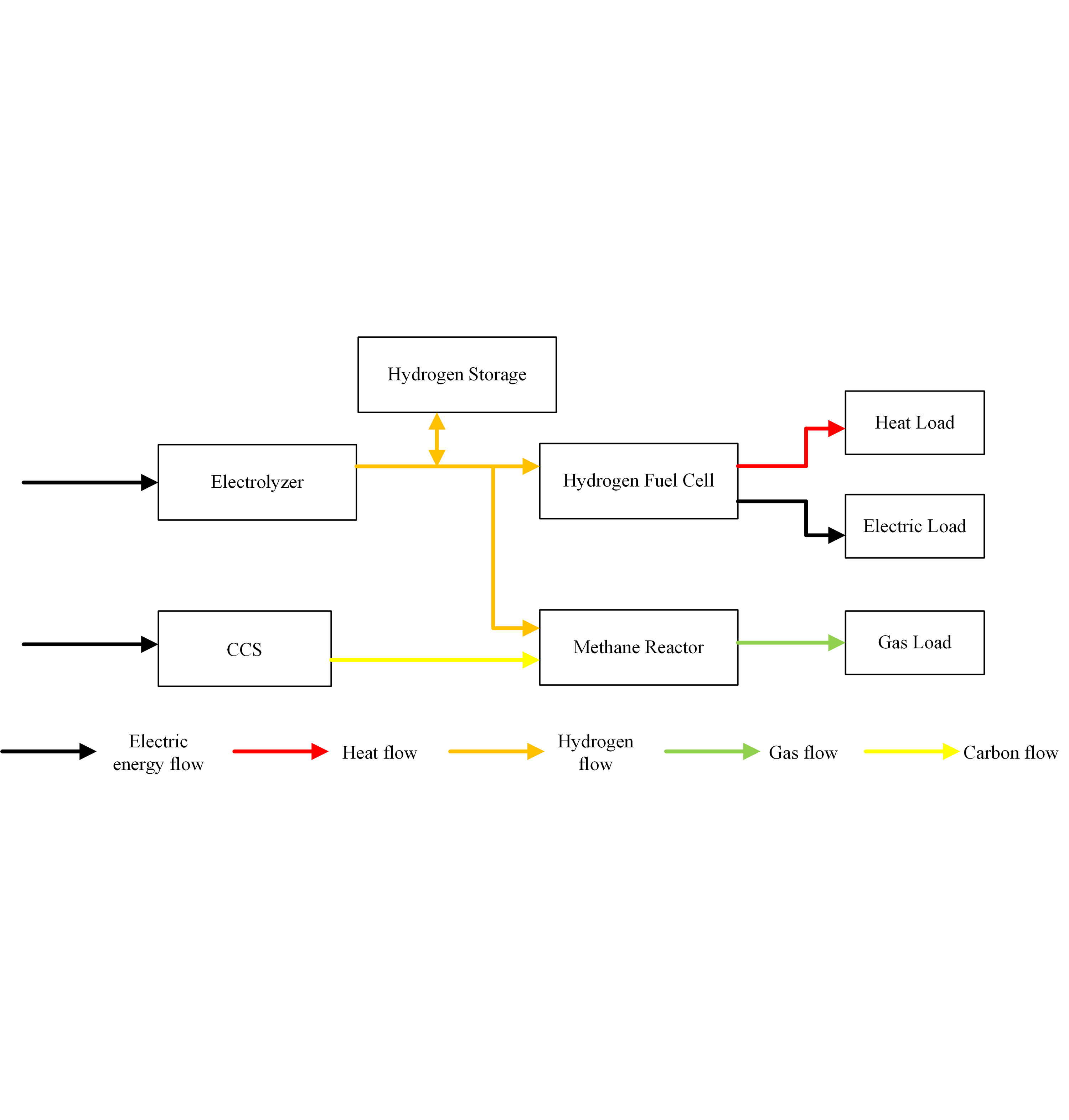

Supplement: S1 Fig — (ZIP) [file pone.0325310.s001.zip › S1 Fig/Fig 3.tif]

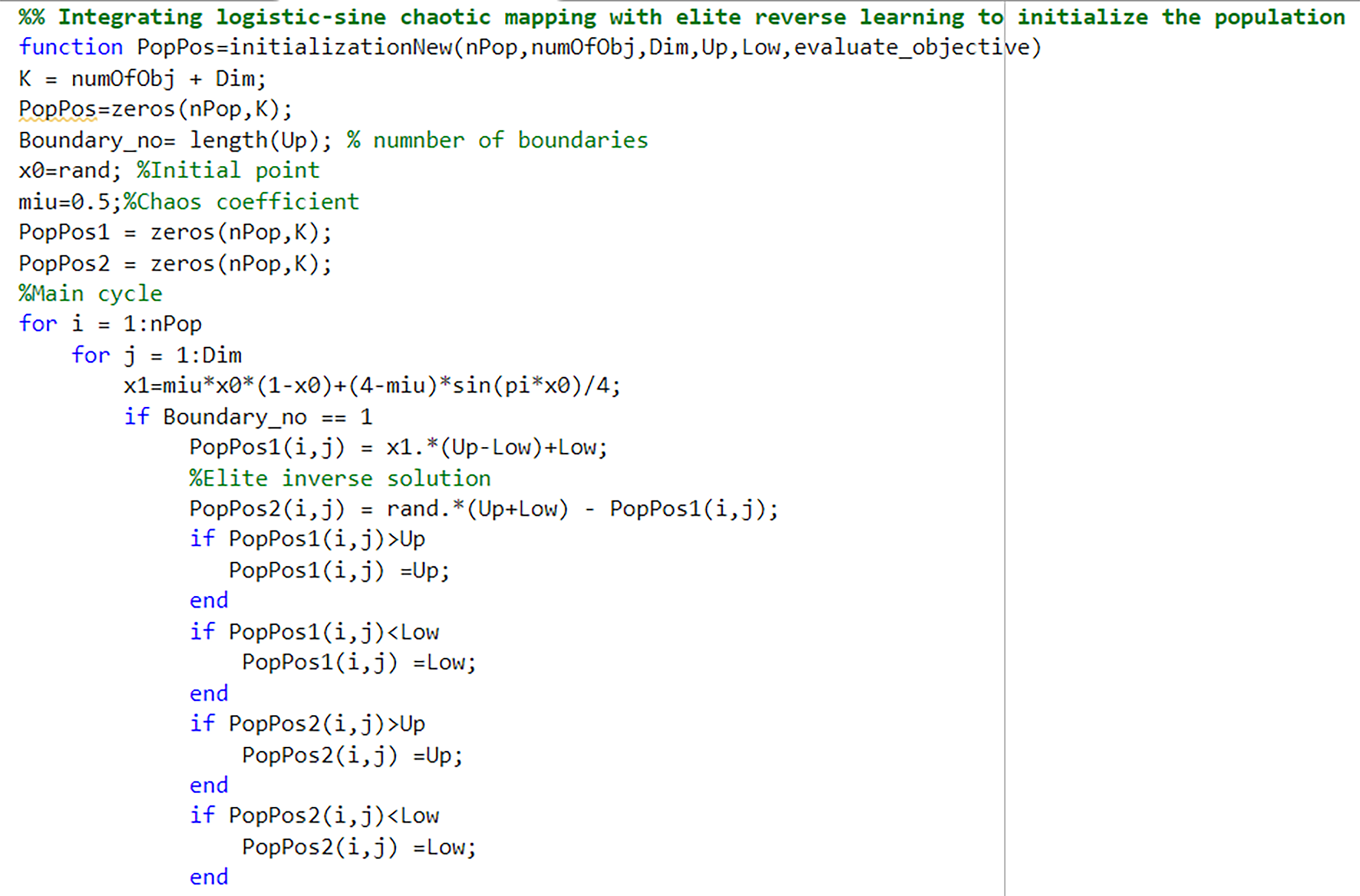

Supplement: S1 Fig — (ZIP) [file pone.0325310.s001.zip › S1 Fig/Fig 4.png]

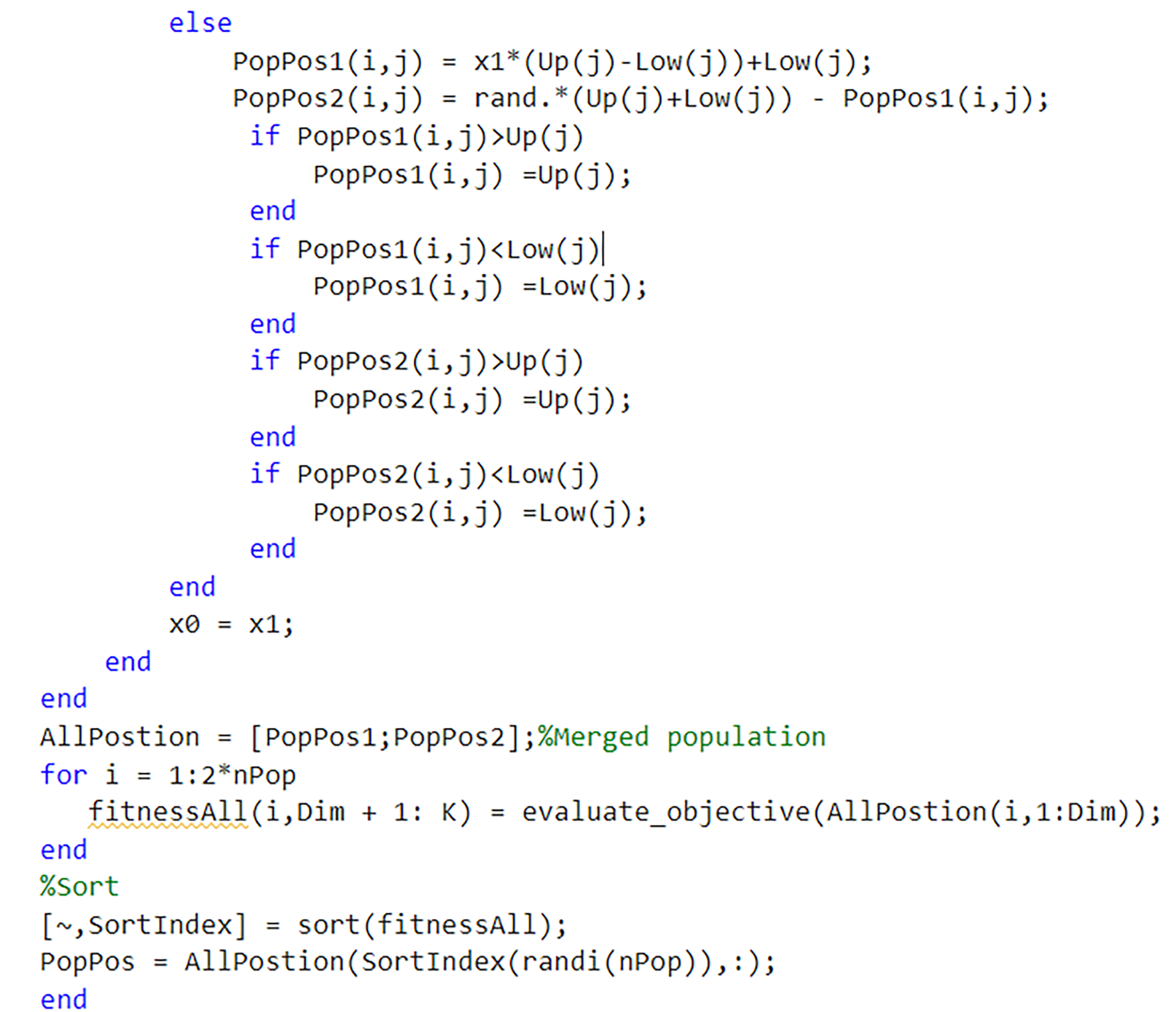

Supplement: S1 Fig — (ZIP) [file pone.0325310.s001.zip › S1 Fig/Fig 5.png]

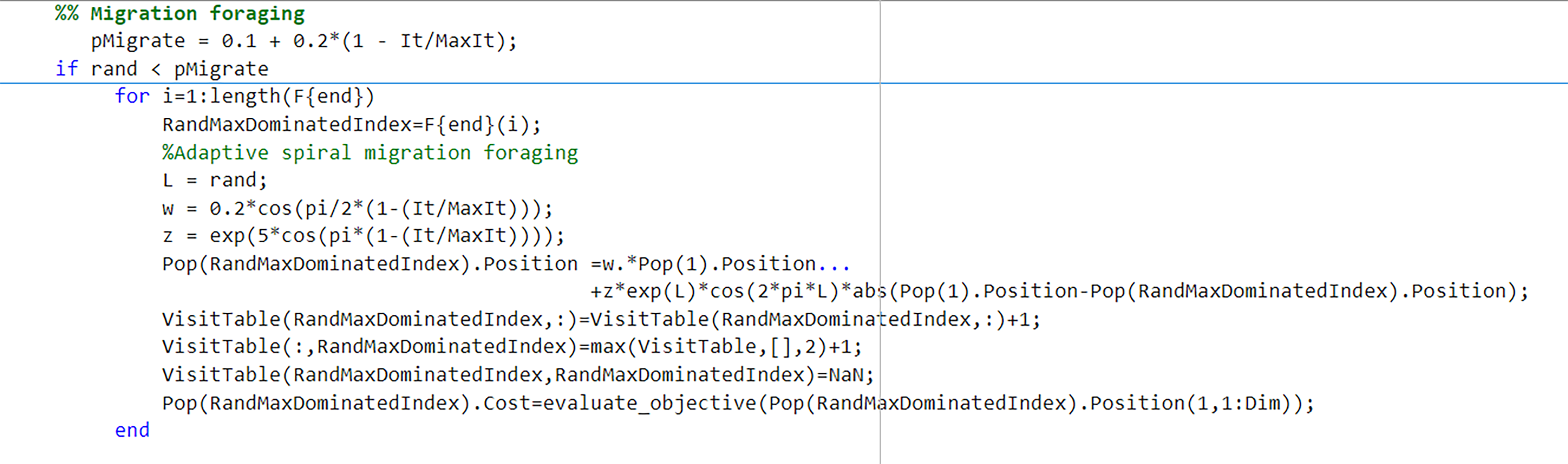

Supplement: S1 Fig — (ZIP) [file pone.0325310.s001.zip › S1 Fig/Fig 6.png]

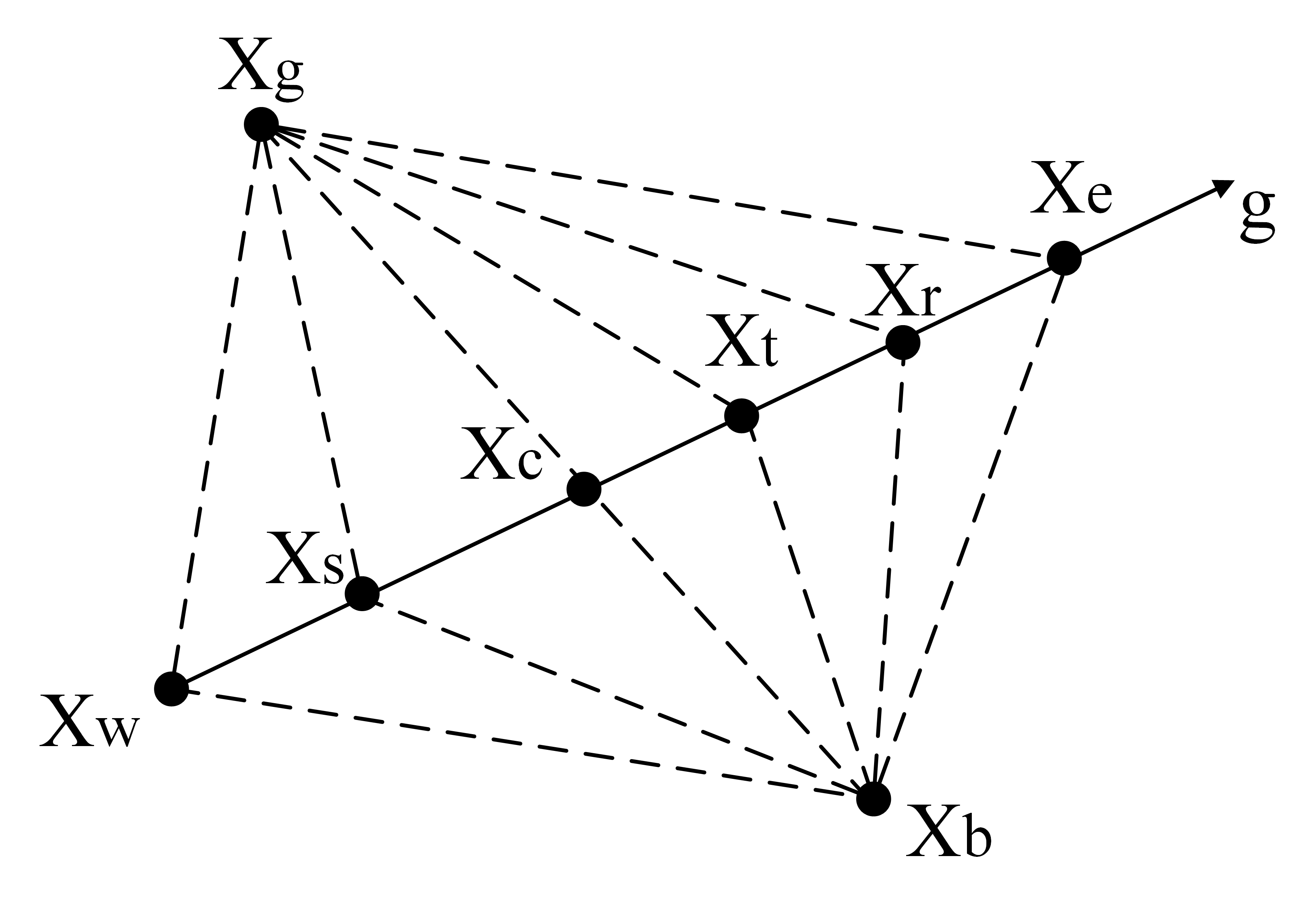

Supplement: S1 Fig — (ZIP) [file pone.0325310.s001.zip › S1 Fig/Fig 7.tif]

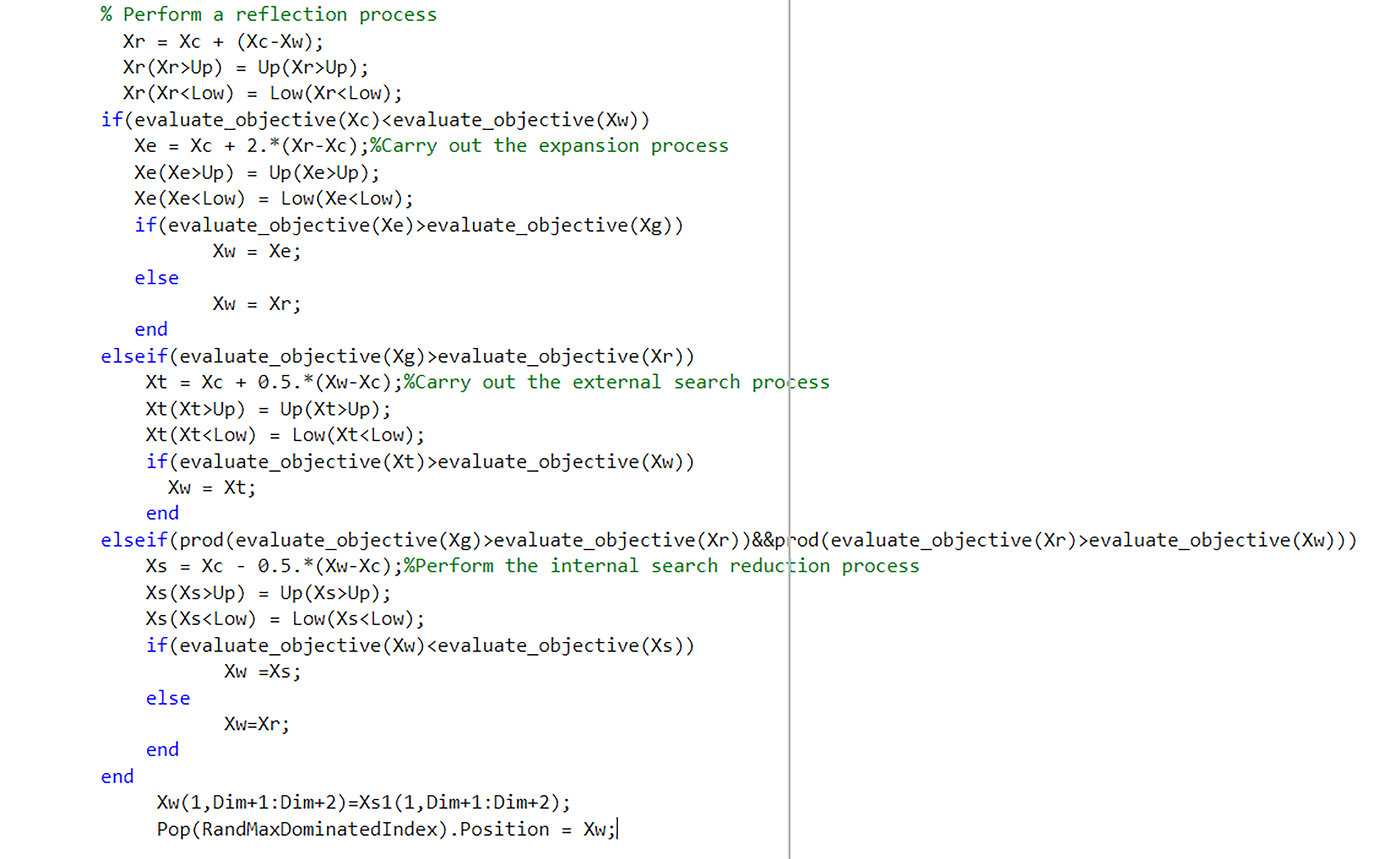

Supplement: S1 Fig — (ZIP) [file pone.0325310.s001.zip › S1 Fig/Fig 8.png]
