# Supplementary material for: Research on optimal scheduling of integrated energy system based on improved multi-objective artificial hummingbird algorithm
Source: PLoS One. 2025 Jun 4;20(6):e0325310. doi: 10.1371/journal.pone.0325310 (PMC12136360; doi:10.1371/journal.pone.0325310)
Supplement: S1 Table — (DOCX) [file pone.0325310.s002.docx]

**Table 1.Equipment parameters**

| Argument | Operation and maintenance cost (Yuan /KW•h) | Argument | Operation and maintenance cost (Yuan /KW•h) |
| --- | --- | --- | --- |
| $C_{ER}$ | 0.02 | $C_{CCS}$ | 0.055 |
| $C_{EB}$ | 0.016 | $C_{CCHP}$ | 0.09 |
| $C_{WHB}$ | 0.01 | $C_{PV}$ | 0.024 |
| $C_{WT}$ | 0.0196 | $C_{ac}$ | 0.06 |

**Table 2. Algorithm performance evaluation index results**

| Test function | Index | MOGWO | MODA | MOAHA | The algorithm in this paper |
| --- | --- | --- | --- | --- | --- |
| ZDT1 | IGD | 0.012984 | 0.024413 | 0.0024394 | 0.0023126 |
|  | Spacing | 0.01317 | 0.26559 | 0.0037197 | 0.0033773 |
| ZDT2 | IGD | 0.0058498 | 0.0050581 | 0.0025225 | 0.0024034 |
|  | Spacing | 0.0079424 | 0.0064633 | 0.0036172 | 0.0034111 |
| ZDT3 | IGD | 0.012518 | 0.027773 | 0.0048794 | 0.0046054 |
|  | Spacing | 0.012763 | 0.010672 | 0.0064069 | 0.0060585 |
| ZDT6 | IGD | 0.0034893 | 0.0040693 | 0.0021258 | 0.0019509 |
|  | Spacing | 0.011178 | 0.12212 | 0.0019194 | 0.0017878 |

**Table 3. System parameters of each unit**

| Argument | Numerical value | Argument | Numerical value |
| --- | --- | --- | --- |
| $P_{CCHP}^{max}$ | 3*MW* | $C_{AC}^{max}$ | 2.5*MW* |
| $R_{WHB}^{max}$ | 3*MW* | $C_{ER}^{max}$ | 1.5*MW* |
| $P_{CCS,e}^{max}$ | 2*MW* | $P_{EL,e}^{max}$ | 2*MW* |
| $P_{MR}^{max}$ | 0.8*MW* | $R_{EB}^{max}$ | 1.5*MW* |
| $P_{HFC}^{max}$ | 0.8*MW* | $\eta_{e}$ | 0.6 |
| $\eta_{loss}$ | 0.05 | $\eta_{h}$ | 1.9 |
| $\eta_{H}$ | 0.95 | $r^{c}$ | 0.4*t/MW•h* |
| $\eta_{c}$ | 2.4 | $\lambda^{c}$ | 0.392*t/MW•h* |
| $r^{e}$ | 1.08*t/MW•h* | $\alpha$ | 250*¥/t* |
| $\lambda^{e}$ | 0.5*t/MW•h* | $g$ | 0.39 |
| $l$ | 2*t* | $\eta_{CCS}$ | 0.85 |
| $\lambda_{CCS}$ | 0.31*MW•h/t* | $\eta_{HFC,h}$ | 0.9 |
| $\eta_{EL}$ | 0.88 | $\eta_{HFC,e}$ | 0.9 |
| $\eta_{MR}$ | 0.55 | $R_{{CH}_{4}}$ | 36*MJ/m^3^* |
| $\rho_{\text{CO}_{2}}$ | 2*kg/m^3^* | $R_{EH}$ | 3600*MJ/MW* |
| $\eta_{EB}$ | 0.9 | $\eta_{ER}$ | 2 |
| $P_{\text{ES}\text{,}\text{e}}^{\text{min}}$ | 0*MW* | $P_{\text{ES}\text{,}\text{e}}^{\text{max}}$ | 1*MW* |
| $P_{\text{ES}\text{,}\text{H}}^{\text{min}}$ | 0*m^3^* | $P_{\text{ES}\text{,}\text{H}}^{\text{max}}$ | 500*m^3^* |
